# Correlations between Negative Symptoms and Cognitive Deficits in Individuals at First Psychotic Episode or at High Risk of Psychosis: A Systematic Review

**DOI:** 10.3390/jcm12227095

**Published:** 2023-11-14

**Authors:** Antonio Melillo, Edoardo Caporusso, Giulia Maria Giordano, Luigi Giuliani, Pasquale Pezzella, Andrea Perrottelli, Paola Bucci, Armida Mucci, Silvana Galderisi

**Affiliations:** Department of Psychiatry, University of Campania “Luigi Vanvitelli”, Largo Madonna delle Grazie, 80138 Naples, Italy

**Keywords:** schizophrenia, psychosis, high risk of psychosis, clinical high risk of psychosis, first-episode psychosis, negative symptoms, cognitive impairment, neurocognition, social cognition

## Abstract

The present review aims to identify correlations between negative symptoms (NS) and deficits in neurocognition and social cognition in subjects with first-episode psychosis (FEP) and at-high-risk populations (HR). A systematic search of the literature published between 1 January 2005 and 31 December 2022 was conducted on PubMed, Scopus, and PsycInfo. Out of the 4599 records identified, a total of 32 studies met our inclusion/exclusion criteria. Data on a total of 3086 FEP and 1732 HR were collected. The available evidence shows that NS correlate with executive functioning and theory of mind deficits in FEP subjects, and with deficits in the processing speed, attention and vigilance, and working memory in HR subjects. Visual learning and memory do not correlate with NS in either FEP or HR subjects. More inconsistent findings were retrieved in relation to other cognitive domains in both samples. The available evidence is limited by sample and methodological heterogeneity across studies and was rated as poor or average quality for the majority of included studies in both FEP and CHR populations. Further research based on shared definitions of first-episode psychosis and at-risk states, as well as on more recent conceptualizations of negative symptoms and cognitive impairment, is highly needed.

## 1. Introduction

Schizophrenia has a multifaceted clinical picture [1] consisting of positive, negative, disorganized, and affective symptoms, as well as neurocognitive and social cognition impairments [2,3]. Among them, since schizophrenia’s early descriptions, negative symptoms and cognitive impairment have been recognized as significant characteristics of the condition which are already detectable during the very early stages of the disease, influencing short- and long-term outcomes [4,5,6,7,8,9,10].

According to the current conceptualization, schizophrenia’s negative symptoms include avolition, asociality, anhedonia, blunted affect, and alogia [11]. While different studies found that these symptoms cluster in two domains, the Motivational Deficit domain (MAP) (including avolition, anhedonia, and asociality) and the Expressive Deficit domain (EXP) (including alogia and blunted affect) [4,5,12,13,14], more recent large-scale studies on multicenter samples have supported a model comprising five factors, one for each individual negative symptom, and a hierarchical model, with the five individual NS constituting first-order factors and the two domains, MAP and EXP, as second-order factors [15,16,17,18]. Negative symptoms often appear before attenuated psychotic symptoms in individuals at high risk of psychosis (HR subjects) [19,20,21,22,23,24] and are associated with a higher risk of conversion to psychosis and worse real-life functioning [9,21,25,26,27,28,29,30,31,32,33,34]. In first-episode psychosis (FEP), the severity of negative symptoms is associated with a poor quality of life, functional recovery and adherence to treatment, and with an increased risk of self-harm [35,36,37,38,39,40,41,42].

The impairment in different domains of cognition represents another important feature of schizophrenia, observed in all stages of the disorder, independently of the severity of the symptoms, in the premorbid and prodromal states, as well as in an attenuated form, in non-affected relatives of subjects with schizophrenia [43,44,45,46,47]. According to the current conceptualization, cognitive impairment in subjects with schizophrenia includes deficits in six neurocognitive (NC) domains: Processing Speed, attention/vigilance (A/V), Working Memory (WM), verbal learning and memory (VeLM), visual learning and memory (ViLM), and reasoning and problem-solving [48,49]. Additionally, social cognition (SC), which involves the mental processes underlying social behavior, such as interpreting other persons’ intentions or emotions, has been identified as an additional separate domain. Persons experiencing their first psychotic episode and those at high risk for psychosis often exhibit broad and enduring cognitive impairment (CI) across several domains, including Working Memory, executive functions (EF), A/V, PS, learning, memory, and SC [50,51,52,53]. CI has been linked to a number of important outcomes in psychotic disorders, such as relapse rates, hospitalization duration, symptom severity, social functioning, vocational functioning (i.e., ability to work or attend school), treatment resistance, and independent living/residential status [46,54,55,56,57,58,59,60,61].

Tracing the boundaries between negative symptoms and cognitive impairments is still a concern. Indeed, both these aspects share strong associations with the functional outcome in people with schizophrenia [4,62,63,64]. However, since many studies have used assessment instruments that include items related to neurocognition and that focus on behavioral manifestations, rather than on internal experiences, for the evaluation of negative symptoms, it is difficult to say whether commonalities between the two dimensions are partly explained by these confounders [8]. An overlap between negative symptoms and CI may also be due to shared underlying mechanisms: an impairment of EF, for instance, might interfere with the goal-directed behavior needed for achieving a reward, which is conceptualized as a pathophysiological mechanism underlying the MAP [5], and poor social cognition might result in or be the result of asociality [5,65]. In addition, the EXP domain of negative symptoms (blunted affect and alogia) might be underpinned by deficits in social cognition and neurocognition. A blunted affect might be due to deficits in emotion identification and discrimination and, more in general, abnormalities in the perception of nonverbal social cues, with a consequent inability to infer meaning from social situations and behaviors and to respond appropriately [8,66], while alogia might result from a poor Verbal Fluency, i.e., from a deficit in the ability to retrieve information from memory, according to the cognitive resource limitation model [8,66].

Several studies have established an association between negative symptoms and cognitive impairment in schizophrenia. Previous research conducted in subjects with schizophrenia has shown that negative symptoms are negatively correlated with neuropsychological performance, including EF, VF, VeLM, A/V, Working Memory, and Processing Speed [9,67,68,69]. Although studies have highlighted a correlation between negative symptoms and different cognitive deficits, several issues still remain unclear, in particular, whether different cognitive domains have distinct associations with the two negative symptom domains or with the five individual negative symptom, or whether general cognitive abilities play a role, as suggested by several studies [70,71,72,73,74,75,76], and whether different confounding factors have an impact on this relationship. In fact, conflicting and divergent findings have been reported regarding the connections between cognitive deficits and the two domains of negative symptoms, i.e., MAP and EXP. Both domains have been associated with impaired EF. EXP has been found to be associated with VF, memory, and symbol coding too [77,78], while MAP has also been related to Working Memory, VF, visual information, and VeLM [79,80,81]. Ventura et al. [82] found correlations between both domains, assessed with the Scale for the Assessment of Negative Symptoms (SANS), and neurocognitive deficits, while, in a large-scale multicenter study conducted by Galderisi et al. [83], neurocognitive deficits correlated weakly with MAP and moderately with EXP, assessed with the Brief Negative Symptom Scale (BNSS). A few studies [84,85] failed to find significant correlations between the two negative symptom domains assessed with the Clinical Assessment Interview for Negative Symptoms (CAINS) and cognitive deficits.

Discrepancies among studies may be due to the use of different methodologies, such as different study designs and the use of different assessment tools for negative symptoms and cognitive deficits. In particular, for the assessment of negative symptoms, the predominant use of first-generation scales, (i.e., scales developed before the NIMH-Negative Symptom Consensus Development Conference) [11] creates potential overlap with both the functioning and cognitive areas [8,18]. Inconsistencies among different studies may also be due to clinical confounding factors, such as chronic antipsychotic medication effects, institutionalization, chronic psychotic symptoms, and poor physical health [86,87]. Therefore, examining the relationships between negative symptoms and cognitive deficits in individuals in their first episode of psychosis or those who are at high risk of developing psychosis can minimize the effects of these potentially confounding factors.

To this end, we carried out a systematic review of the literature, focusing on the prodromal and early stages of the pathology to concentrate on a study population more homogeneous with respect to the stage of the illness and the psychopathological condition, and to study negative symptoms and cognitive dysfunctions in a phase of the illness where the clinical picture is not yet conditioned by pharmacological treatment.

In particular, the study aimed to:

- Summarize the available evidence on the correlations between negative symptoms and dysfunctions in neurocognition and social cognition in subjects with first-episode psychosis or who are at-risk.
- Identify possible methodological limitations especially relevant to the non-standardized and heterogeneous conceptualization of negative symptoms and the use of different scales for their evaluation.

## 2. Methods

The reporting of this systematic review was guided by the standards of the Preferred Reporting Items for Systematic Reviews and Meta-analyses (PRISMA) We conducted a systematic review of the scientific literature published in the last 17 years (1 January 2005–31 December 2022) in the following databases: PubMed, Scopus, and PsycInfo. The following combination of search terms was used: (“psychosis” OR “first-episode psychosis” OR FEP OR FES OR “first-episode schizophrenia” OR “Ultra-High Risk” OR UHR OR “Clinical High Risk” OR HR) AND (“negative symptoms” OR avolition OR apathy OR anhedonia OR alogia OR asociality OR amotivation OR “social withdrawal” OR “blunted affect” OR “affective flattening” OR “persistent negative symptoms” OR “predominant negative symptoms” OR “prominent negative symptoms” OR “primary negative symptoms” OR “deficit schizophrenia” OR “lack of motivation”) AND (neurocognit* OR cognit* OR memory OR “verbal learning” OR “verbal memory” OR “visual learning” OR “visual memory” OR “visual-spatial learning” OR “visual-spatial memory” OR “working memory” OR attention OR vigilance OR “PS” OR “speed of processing” OR reasoning OR “problem solving” OR “executive function*” OR “social cognition” OR “emotion perception” OR “emotion recognition” OR “theory of mind” OR “social knowledge”). Three investigators independently screened all articles for eligibility based on titles and abstracts, and then they proceeded to read the full text. Discrepancies in the selection of suitable articles were discussed by the entire research group and were resolved through discussion and consensus.

Inclusion criteria were: (1) study sample including subjects with first episode of nonaffective psychosis or subjects at high risk of psychosis; (2) availability of correlations between at least one domain of cognition and negative symptom severity; (3) publication date not earlier than the last 15 years; and (4) English language. Exclusion criteria for FEP studies were: subjects > 65 years old, >1 psychotic episode, >5 years between psychosis onset and study inclusion. Exclusion criterion for HR studies was: subjects’ age > 40 years. Studies that included subjects with affective psychoses were included only if subjects with non-affective psychoses were included and relevant data reported separately.

For studies that met the inclusion criteria, data extraction was performed independently by two independent researchers and included: study authors, year of publication and design, sample size, sample clinical and demographic characteristics (including age, gender, years of education, diagnoses and diagnostic procedures, and, when appropriate, duration of illness, duration of untreated psychosis and pharmacological treatment at the time of study enrollment), NS and CI assessment methods, statistical analysis methods, and main findings examining the relationship between NS and CI. The risk of bias and the methodological quality of the retrieved studies was assessed following the Joanna Briggs Institute’s (JBI) Critical Appraisal tools by two independent researchers. Depending on the checklist scores, studies were categorized as having low, average, and good methodological quality. Disagreements on each item of the applied checklists were resolved through discussion with a third reviewer.

The retrieved evidence was organized in nine categories referring to associations between NS and eight cognitive domains. In particular, alongside the seven cognitive domains actually recognized as the most frequently impaired in subjects with schizophrenia (Processing Speed, A/V, EF, Working Memory, VeLM, ViLM, and SC) and in line with the meta-analysis by De Gracia Dominguez et al., 2009 [88], we chose to consider Verbal Fluency as a separate domain from processing speed, given the evidence on the impairment of this area and its relationship with NS in subjects with schizophrenia [89,90,91]. When possible, heterogeneity among retrieved evidence was examined in the light of methodological differences and risk of bias.

## 3. Results

### 3.1. Search Results

**Figure 1:**
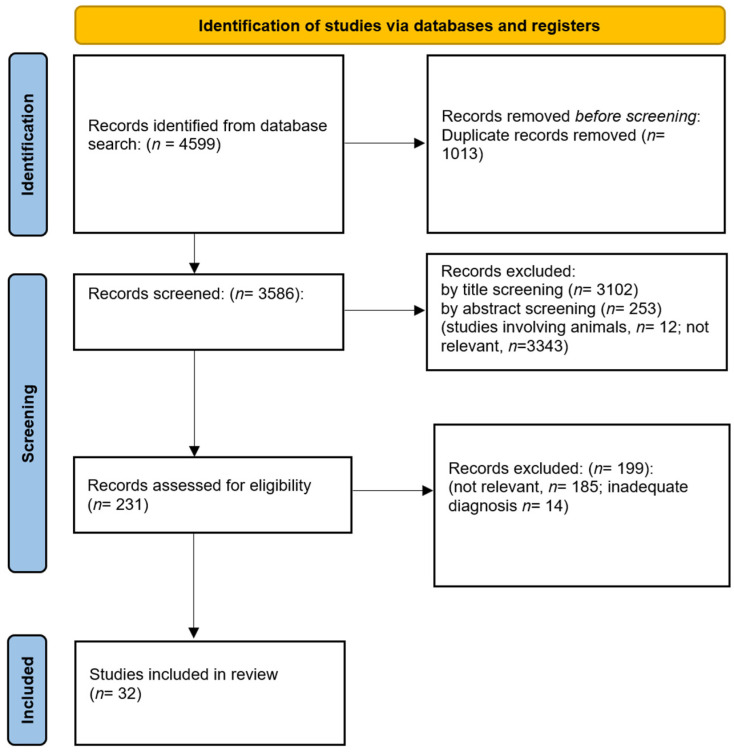
PRISMA flowchart of the selection process.

Database searching produced 4599 results. After the removal of duplicates, a total of 3586 articles were included. Two researchers (A.Me. and E.C.) individually reviewed the titles and abstracts of all the articles to determine their suitability. Subsequently, they proceeded to read the full text of these articles. Any disagreements regarding the selection of suitable articles were resolved through group discussion and consensus. After title and abstract screening, a total of 231 articles were assessed for eligibility. Of these 231 articles, 199 were excluded because they were not relevant to the topic (*n* = 185) or they included subjects with a diagnosis that did not meet our criteria (e.g., affective disorders with psychotic symptoms) [52,91,92,93,94,95,96,97,98,99,100,101,102,103]. Therefore, a total of 32 articles met our criteria and were included in the systematic review (see Figure 1 for the PRISMA flowchart of the selection process). In total, 21 studies concerned subjects with first-episode psychosis; 14 had a cross-sectional design, 6 were longitudinal studies, and 1 performed a network analysis. Additionally, 11 studies targeted the high-risk population; eight were cross-sectional and three were longitudinal studies. In total, data from 3086 FEP subjects and 1732 HR subjects were analyzed.

For a summary of the data extracted from the included studies and for the assessed quality please refer to Tables S1 and S2.

### 3.2. Risk of Bias and the Methodological Quality of the Retrieved Studies

Among the studies on first-episode psychosis, nine were categorized as being of good methodological quality [104,105,106,107,108,109,110,111,112,113], six studies as being of an average methodological quality [78,114,115,116,117,118], and five as being of poor methodological quality [68,119,120,121,122]. In relation to studies on the clinical high-risk population, only one study was categorized as being of good methodological quality [123], while three studies were categorized as being of average methodological quality [31,124,125], and seven studies as being of poor methodological quality [126,127,128,129,130,131,132].

In the case of the studies on FEP subjects, studies with average or poor methodological qualities often did not adequately describe study subjects and settings, particularly in regard to the pharmacological treatment, age of onset, duration of untreated illness, and duration of untreated psychosis (see Section 3.3). In the case of studies on HR subjects, most studies did not adequately describe recruitment strategies, clinical features, eventual co-existing diagnoses, pharmacological treatment, or educational level (see Section 3.4). More in general, the level of evidence of studies on both populations was hindered by potential confounding factors and by inappropriate methodologies for the assessment of NS, as we will describe further in detail in Section 3.5.

### 3.3. Sample Inclusion/Exclusion Criteria and Features of FEP Subjects

Studies including FEP subjects applied different definitions of FEP, with most studies using the criterion of first contact with mental health services for psychotic symptoms, and some studies setting a maximum time interval (from 2 weeks to 2 years) between the first prescription of antipsychotic medication or hospitalization due to psychotic symptoms. Other frequent inclusion criteria were an IQ > 70, the ability to provide consent, a diagnosis of a schizophrenia spectrum disorder (schizophrenia, schizoaffective disorder, schizophreniform disorder, paranoid schizophrenia, undifferentiated schizophrenia, and schizotypal disorder), delusional disorder, brief psychotic disorder, or psychosis not otherwise specified. The main exclusion criteria were age (minimum age most frequently set at 18 years old and the maximum age at enrollment between 34 and 65 years), history of neurological disorders, severe head injury, intellectual disability, and psychosis secondary to substance abuse, alcohol, or general medical conditions. The demographic and clinical features of the included studies are summarized in Table 1(a). In relation to sample size, included studies showed significant heterogeneity, as the largest sample was 323 subjects and the smallest was 20. Out of 3086 subjects included in the present review, 1508 received a diagnosis of schizophrenia; 75 with a schizoaffective disorder, 290 with a schizophreniform disorder, 227 with a delusional disorder, 175 with a brief psychotic disorder, 451 with a schizophrenia-spectrum disorder not otherwise specified, and 360 with a first episode of a psychotic disorder not otherwise specified. Gender was almost balanced, with a small preponderance of males (53.7%). The mean age ranged from 21.4 to 38.3 years and the mean age of psychosis onset between 21.9 and 36.6 years, while the duration of untreated psychosis was between 1 and 108 months.

Details on pharmacological treatment were specified in only 10 out of the 21 included studies [68,105,106,108,111,114,116,117,119,122]. Most subjects were taking second-generation antipsychotics (*n* = 912), while a minority were treated with first-generation antipsychotics (*n* = 142). Also, the duration of treatment varied from studies that included antipsychotic-naïve subjects [111,114,116] to studies that included subjects with less than two [111], three [112], four [104,110], or six weeks [114,117] of treatment at the time of enrolment. Few studies included subjects with a longer duration of treatment (4 to 12 months) [106,107,109,115].

### 3.4. Sample Inclusion/Exclusion Criteria and Features of HR Subjects

The 11 studies focusing on HR populations applied the following criteria to define the risk of psychosis: six studies applied the Structured Interview for Psychosis-Risk Syndromes (SIPS) criteria [124,126,127,128,130,132], two studies applied The Comprehensive Assessment of At-Risk Mental States (CAARMS) criteria [123,131], one study used both the Structured Interview for Psychosis-Risk Syndromes (SIPS) and the CAARMS to enroll subjects [125], one study applied the Basel Screening Instrument for Psychosis (BSIP) [129], and one applied the following operational criteria, (a) Brief Limited Psychotic Symptoms (BLIPS), (b) attenuated psychotic symptoms assessed by using the Brief Psychiatric Rating Scale-Expanded, and (c) family risk with reduced function, which was defined as having both a first-degree family member with a psychotic disorder or schizotypal personality disorder and functional impairment defined as a decrease of at least 30 points on the Global Assessment of Functioning scale [31].

The main inclusion criterion for HR studies was the positivity to the HR criteria (as assessed through SIPS or CAARMS) among help-seeking individuals. In relation to age, most studies enrolled subjects with an age range of 16–18 and 35–40, with the exception of one study, which enrolled adolescent volunteers and set a maximum age of 18 [128]. The main exclusion criteria were the previous diagnosis of psychotic disorders or previous psychotic episodes, IQ < 70, and past or current history of a neurological disease. In addition, 3 out of 11 studies explicitly set the presence of past or current substance abuse as a further exclusion criterion [31,125,132].

Data from a total of 1732 subjects were included. Sample sizes varied significantly and were between 675 (the largest sample) and 45 subjects (the smallest one). The demographic and clinical features of subjects participating in the included studies are summarized in Table 1(b). Subjects were predominantly males (60.6%), with a mean age range between 15.5 and 26.9 years. Details of the eventual previous psychiatric diagnoses were available for a minority of included subjects (affective disorder = 48; anxiety disorder = 50; substance-induced psychotic disorder = 9; somatoform disorder = 3; eating disorder = 2; adjustment disorder = 2; personality disorder = 15). Only 6 out of the 11 included studies [31,123,124,129,130,132] reported information on the treatment at the time of the enrolment: most subjects were not currently under antipsychotic medication (*n* = 594) or were antipsychotic-naïve (107), while a small percentage of subjects were treated with antipsychotic medication (SGA = 34, antipsychotics not otherwise specified = 44).

**Table 1:** (**a**,**b**) Demographic and clinical features of the included samples.

(**a**). **Demographic and Clinical Features of FEP Subjects**	**FEP = 3086**
**Diagnosis, *n*. of subjects**	SCZ = 1508 SCZ-A = 75 Schizophreniform = 290 DD = 227 BPD = 175 SSD-NOS = 451 PD-NOS = 360
**Psychopharmacological treatment, *n*. of subjects**	SGA = 912 FGA = 142 Anticholinergics = 50 Antidepressants = 13 Antidepressants + benzodiazepines = 1 Antipsychotic-naïve = 179 *
**Mean age of onset range across studies (min.–max.)**	21.9–36.6 ** years
**Mean duration of untreated psychosis/illness across studies (min.–max.)**	1–108 *** months
**Mean age across studies (min.–max.)**	21.4–38.3 years
**Gender, *n*. of male subjects (% of male subjects)**	1656 (53.7%)
**Mean education across studies (min.–max.)**	10.2–12.6 years
(**b**). Demographic and clinical features of HR subjects	**HR = 1732**
**Gender, *n*. of male subjects (% of male subjects)**	1051 (60.6%)
**Diagnosis, *n*. of subjects**	AD = 48; Anxiety disorder = 50; SI-PD = 9; Somatoform disorder = 3; ED = 2; Adjustment disorder = 2; Personality disorder = 15
**Mean age across studies (min.–max.)**	15.5–26.9 years
**Mean education across studies (min.−max.)**	10.3–14.3 years #
**Psychopharmacological treatment, *n*. of subjects**	Antipsychotic, NOS = 44 SGA = 34 Antipsychotic-naïve = 107 Antidepressants = 52 Mood stabilizers = 11 Benzodiazepines = 11 Psychostimulants = 3 Not currently under antipsychotic medication = 594 ##

Demographic and clinical features of the included samples. AD = affective disorder; DD = delusional disorder; BPD = brief psychotic disorder; ED = eating disorder; FEP= first-episode of psychosis; FGA = first-generation antipsychotics; HR = Clinical high-risk subjects; PD-NOS = Psychotic Disorder—not otherwise specified; SI-PD = substance-induced psychotic disorder; SGA = second-generation antipsychotics; SSD-NOS = Schizophrenia Spectrum Disorder, not otherwise specified; SCZ = Schizophrenia; SCZ-A = schizoaffective disorder. * Available in 9/21 studies; ** available in 6/21 studies; *** available in 14/21 studies. # Available in 6/11 studies; ## available in 6/11 studies.

### 3.5. NS Assessment

#### 3.5.1. Assessment of Negative Symptoms in FEP Subjects

NS in FEP subjects were assessed almost exclusively with first-generation rating scales (Table 2). In particular, eight studies used the Positive and Negative Syndrome Scale (PANSS), ten used the SANS, and one study applied both [105]. Out of the nine studies that used the PANSS, six used the entire negative symptom subscale [68,105,110,118,119,122], one study [121] added general psychopathology items to the PANSS NS subscale (items G13—avolition—and G16—active social avoidance), and one used a nine-item negative factor composed of the NS subscale, excluding items N5 and N7 and including the items G7, G13, G15, and G16 [116]. Only one study [109] applied the PANSS NS subscale excluding the items N5 and N7 in accordance with the EPA guidance on the topic [4]. Of the 11 studies assessing NS with the SANS, two used the entire scale including, therefore, the attention subscale [105,117], four excluded the attention subscale from the total score [104,111,112,114], one employed the SANS excluding aspects not conceptualized as negative symptoms, such as attention, inappropriate affect, and poverty of the content of speech [107], in accordance with the EPA guidance on the topic [4], and four used a model comprising the two domains, i.e., the MAP (Avolition–Apathy and Anhedonia–Asociality subscales) and the EXP (Blunted affect, including, also, the inappropriate affect), and the alogia subscale (sometimes including the poverty of content of speech item) [107,108,113,115]. In summary, most studies assessed negative symptoms with first-generation rating scales also including aspects that are in overlap with cognitive impairment, such as “difficulty in abstract thinking” (for PANSS) and the attention subscale or “poverty in content of speech” (for SANS), while only two studies [107,109] evaluated NS according to their current conceptualization. Other assessment instruments were the Brief Psychiatric Rating Scale (BPRS) [120] and the High Royds Evaluation of Negativity Scale (HEN) [78].

#### 3.5.2. Assessment of Negative Symptoms in HR Subjects

Seven out of eleven studies carried out in HR subjects used rating scales specifically developed for this population [125,126,127,128,130,131,132], while three studies used the SANS [31,123,129] (Table 3). Most studies used the SIPS, which includes social anhedonia, avolition, the expression of emotion, the experience of emotions and self, ideational richness, and also occupational functioning; the last two aspects are not conceptualized as negative symptoms, and the latter one clearly overlaps with the role of functioning.

### 3.6. Assessment of Cognitive Impairment in FEP and HR Subjects

The included studies used different assessment tools to analyze the cognitive functions in FEP and HR subjects. Some studies employed comprehensive test batteries to define neurocognition: three used the MATRICS Consensus Cognitive Battery (MCCB) [111,112,127], two used the Brief Assessment of Cognition in Schizophrenia (BACS) [114,123], one used the Wechsler Memory Scale (WMS) [104], one used the Wechsler Adult Intelligence Scale-III version (WAIS-III) [118], and one used the Cambridge Neuropsychological Test Automated Battery (CANTAB) [122]. Most studies, however, used individual tests to evaluate each cognitive function, with a minority of studies focusing on single functions. Below, we will report those most frequently used to evaluate the impairment in cognitive domains.

For the evaluation of the processing speed, the Trail Making Test and the WAIS Digit Symbol Coding Test were often used [31,111,116,117,118,124,125,128]. For the assessment of Verbal Fluency in most cases, the category verbal fluency test was used. Attention was assessed in most cases with the Continuous Performance Test [111,112,116,117,118,127], and working memory by the Digit Span subtest of the WAIS-R or WAIS-III scale [31,106,107,108,113,118,125] or the MCCB Letter Number Span [111,112,127]. Verbal learning and memory was assessed by the Rey’s Auditory Verbal Learning Test [31,68,117,118] or the MCCB’s Hopkins Verbal Learning Test [111,112,127]. Visual learning and memory was assessed in most cases with the Brief Visuospatial Memory Test-R derived from the MCCB [111,112,118,127]. Reasoning and Problem Solving were mostly assessed with the Neuropsychological Assessment Battery Mazes [111,127]. Social cognition and its subdomains were assessed using different tests: emotional processing with the MCCB MSCEIT test [111,127], social perception with the Awareness of Social Inference Test (TASIT) [114] and the Social Cue Recognition Test (SCRT) [121], and the theory of mind (ToM) with the Hinting Task Test [118].

Please refer to Table S3 for a summary of the applied assessment tools.

### 3.7. Correlations between NS and Cognitive Functions in FEP Subjects

Please refer to Table 4 for a summary of the studies that investigated correlations between the negative symptom total score and cognitive impairment. For a detailed summary of the data extracted from each included study, please refer to Table S1.

In summary, studies investigating the relationship between NS and impairment in different cognitive domains in subjects at the first psychotic episode report a relationship between NS severity and deficits in EF [107,109,116,117,122]. Evidence relevant to the relationship with reasoning and problem solving is regarded as poor [111,118]. Furthermore, it seems that the persistence of negative symptoms influences the EF performance, since two studies reported significantly lower scores in EF tasks in subjects with persistent negative symptoms (PNS) compared to subjects without PNS [106,109]. In addition, included studies reported an absence of a correlation between NS and ViLM [68,111,117,118,122] and the presence of a correlation with ToM deficits [112,114,115]. The evidence on the relation between NS and the Processing Speed, VF, A/V, Working Memory [68,104,108,117,118,119,122], VeLM [68,117,118], and global social cognition scores [114,118,121] was inconsistent, and no final conclusion can be drawn. The use of different assessment instruments (PANSS, SANS) and different criteria (inclusion or exclusion of the attention subscale for SANS; inclusion or exclusion of difficulty in abstract thinking and stereotyped thinking for PANSS) in evaluating negative symptoms might have led to these inconsistent findings [68,109,111,116,117].

It is also interesting to note that in many studies, the total score of negative symptoms did not correlate with the impairment of any particular cognitive domain [116,118] and, in other studies, negatively correlated with the overall cognitive scores.

The evidence on the correlations between cognitive domains and individual NS domains was sparse and heterogeneous. Verbal Fluency correlated with the EXP domain as assessed by the HEN [78] or PANSS [113], but this relationship was not confirmed in a study applying SANS [107]. In relation to Working Memory, one study reported a significant negative correlation between the SANS alogia subscale [111] and Working Memory, while another study did not find any significant correlation between Working Memory and the two negative symptom domains [113].

ViLM correlated with the EXP domain, as assessed through the HEN scale [78]. However, another study did not find any statistically significant association between either EXP or MAP as assessed by the SANS and ViLM [108].

Correlations were reported between EF and either the Expressive deficit, assessed through the HEN scale [78] and SANS subscale [105], or the Motivational Deficit, assessed by the SANS [113]. ToM deficits correlated in particular with the EXP domain [112,114,115] while Bliksted and colleagues [114] found that global social cognition correlated with both alogia and anhedonia–asociality.

### 3.8. Correlations between NS and Cognitive Functions in Subjects at High Risk of Psychosis

Please refer to Table 5 for a summary of the number of studies that investigated correlations between the negative symptom total score and cognitive impairment in HR subjects. For a detailed summary of the data extracted from the included studies please refer to Table S2.

To summarize, in subjects at risk of developing psychosis, studies that investigated the relationship between NS and impairment in different cognitive domains [31,127,128,130], although few and of poor-to-average methodological quality, suggest a relationship between NS severity and deficits in the processing speed, attention and vigilance [31,127,129,130], and working memory [31,127,129], while the relationship between NS severity and VeLM, supported by different studies [128,129,130,132], was not confirmed when excluding aspects related to neurocognition, such as the SANS attention subscale [31]. As found in the FEP population, and also in HR subjects, NS severity seemed not to correlate with ViLM impairment [124,128,130], but the quality of evidence was poor for most studies [128,130]. Finally, our literature search retrieved few, inconsistent, and potentially biased data on the relationship between NS with VF [125,127,129], EF [31,129,130], and SC [126,127,131].

The inconsistency across studies might be in part explained by the inappropriate assessment of negative symptoms, as included studies considered, as negative symptoms, aspects that are related with functioning (item N6, the “occupational functioning” of the SIPS scale), with cognitive impairment (SANS item, “poverty in content of speech”), or with disorganization (SANS item, “inappropriate affect”). Particularly in relation to the SANS item “poverty in content of speech”, this may cause a significant overlap between NS and Verbal Fluency [129] and potentially bias the results. In addition, in many studies, global NS scores did not specifically relate to the impairment of a particular cognitive domain. In the study of Gerritsen and colleagues [127], SIPS NS correlated not only with A/V deficits, but also with deficits in Working Memory and SC, suggesting that negative symptoms might influence the performance in attention and vigilance, working memory, and social cognition tasks, through deficits in the verbal Working Memory and Processing Speed, as the use of these cognitive domains was necessary for all applied tasks [127]. Similarly, in the study of Üçok and colleagues [31], NS, in particular blunted affect (including, however, also inappropriate affect) and alogia (also including, however, poverty in the content of speech), correlated negatively with the performance not only in Working Memory, but also in A/V and Processing Speed tasks, and the Processing Speed additionally correlated with SANS avolition and anhedonia–asociality.

Few and inconsistent data were retrieved on the relationship between individual NS domains and CI. One study reported that PS negatively correlated with SIPS social anhedonia, the reduced expression of emotions, and the avolition item (which, however, correlated also with A/V and reasoning and problem solving scores as well as with a global neurocognition score) [130]. Verbal Fluency was found to correlate in particular with the EXP domain, and more precisely with the SANS alogia subscale [129] and the SIPS Decreased expression of emotion [130].

In relation to VeLM deficits [129,130,132], in Vargas et al., 2018, social anhedonia and avolition, evaluated with SIPS, were reported to be strongly associated with VeLM impairment [132]. This association, however, was disconfirmed by a second study, where the VeLM scores correlated only with the SIPS item “reduced ideational richness”, which correlated with each considered cognitive domain score [130]. Data on the correlations between ViLM and individual NS domains are limited to this single study, where the SIPS item reduced ideational richness negatively correlated with the ViLM scores [130]. In relation to EF impairment, two studies reported a correlation between EF and the EXP domain evaluated by the SANS [31] or between EF and both the MAP and EXP domains evaluated by the SIPS [130]. As to Reasoning and Problem Solving deficits in HR subjects, one study [130] reported a significant correlation with SIPS Social Anhedonia, Avolition, and the Decreased Expression of Emotions; however, in this study [130], all these negative symptoms domains correlated with the Processing Speed and global neurocognition scores too. This study also reported that global NS severity correlated with PS, A/V, and VF.

## 4. Discussion

The first goal of the present review was to explore the relationship between NS and neurocognitive and social cognition deficits in subjects with first-episode psychosis or who were at risk of psychosis. The retrieved literature shows a complex and inconsistent picture, which limits the possibility to draw firm conclusions.

In relation to FEP subjects, some consistent findings were found in relation to the association between NS severity and ViLM, Reasoning and Problem-Solving, EF, and ToM deficits. Specifically, no significant correlation was found between NS and ViLM [68,111,117,118] and Reasoning and Problem Solving [111,118], while a significant relationship emerged with EF deficits in general (not limited to Reasoning and Problem Solving) [107,109,116,117,122] and ToM [112,114,115,120].

The link between negative symptom severity and the impairment in executive functions was also supported by the evidence of these deficits, in particular, deficits in cognitive flexibility and concept formation in subjects with primary and persistent negative symptoms (deficit schizophrenia, DS), as compared to those without DS [133,134,135], suggesting that this link may be more evident when the role of confounding factors (i.e., other psychopathological dimensions, different clinical courses, and medications) is ruled out. This relationship might be interpreted in the light of the hypothesis that negative symptoms, in particular deficits in motivation, might be subtended by a general impairment in decision making and the executive control of behavior; however, this hypothesis needs further investigation [8].

Moreover, the included studies [112,114,115,120] showed that NS are correlated with ToM impairment in FEP subjects. Indeed, NS severity has been linked strongly to ToM deficits in adult subjects with a diagnosis of schizophrenia spectrum disorder, and a recent systematic meta-analysis reported a significantly stronger association between NS and ToM, compared to associations of ToM deficits with other symptoms and clinical features, with the exception of the neurocognitive and disorganization dimensions [136]. In addition, the association between NS and ToM seems to be stronger in young subjects as well as in those with an early onset of the disease, hence arguing that earlier psychopathological progression may prevent the physiological development of ToM abilities, which, in healthy conditions, would continue to develop until young adulthood [137,138]. The aforementioned meta-analysis [136] failed, however, to demonstrate an association between individual NS domains and ToM deficits. Similarly, the evidence on these relations analyzed in the present review is still sparse and inconsistent [112,114,115,121], and no final conclusions can be drawn for FEP subjects either.

On the other hand, discrepant and inconsistent findings emerged on the relationship between NS and several other cognitive areas, including the Processing Speed [68,109,111,116,117], Verbal Fluency [68,117,118,119], A/V [68,116,117,122], Working Memory [68,104,108,117,118,119,122], VeLM [68,117,118], and SC (when considered as a global score) [114,118,121]. The inconsistency of the findings, as we will further discuss in detail, might be explained by the significant heterogeneity among studies in relation to the clinical characterization of samples and other methodological issues. For instance, samples differed across studies in terms of illness phase and duration, duration of untreated psychosis [139], as well as duration, type, and dosage of antipsychotic treatment. Importantly, as supported also by a recent meta-analysis, the antipsychotic treatment might represent a confounding factor since it might significantly impact neurocognitive performances [140]. In addition, there was significant heterogeneity in relation to how NS were conceptualized and assessed, as shown by the frequent inclusion of aspects not relevant to the current negative symptom construct, which might have resulted in misleading significant associations, due to the overlap with those irrelevant items with other investigated domains (e.g., cognition and real-life functioning) [68,110,117,118,119,122,129].

Overall, some of the findings reviewed in the present paper are only partially consistent with those reported by a previous meta-analysis on a similar topic conducted by Gracia Dominguez and colleagues [88]. However, it should be stated that trying to compare our results with those of the previous meta-analysis could be misleading, due to methodological and sampling differences. Indeed, between the present review and the previous meta-analysis, there is not only a temporal distance, but also a focus on different populations (subjects with chronic psychosis in Gracia Dominguez, FEP, and UHR in the present review). Nonetheless, when comparing the results, the previous meta-analysis showed that NS did not correlate with executive control and Working Memory, while they correlated with deficits in reasoning and problem solving, VF, PS, A/V, as well as ViLM and VeLM. Interestingly, ViLM and VeLM impairment has been linked to negative symptoms, in particular to alogia, as well as to anhedonia, while deficits in visual and verbal memory have been linked to difficulties in retrieving past pleasant experiences, which would impair individuals’ ability of rating past or anticipated experiences as enjoyable [141,142]. However, in our review of the literature, VeLM, but not ViLM, deficits correlated with NS severity [68,117,118], supporting the hypothesis that probably the inability to retrieve from memory and, thus, to report past experiences/feelings as pleasant, could be conceptualized as an impairment of VeLM [143], rather than ViLM. However, the overall retrieved evidence from the present review is still not very robust and further studies are needed in order to confirm this hypothesis and to disentangle the relative contribution of individual negative symptoms.

In relation to the at-risk populations, evidence on the relationship between NS and cognitive deficits is still sparse, as the screening of the current literature according to our inclusion and exclusion criteria only retrieved 11 studies. Nonetheless, among the most robust findings in HR subjects, no significant correlation was found between NS and ViLM (although only two studies investigated this relationship) [128,130], while NS severity significantly correlated with the Processing Speed [31,128,130], A/V [31,127,129,130], Working Memory [31,127], and VeLM impairment [128,129,130,132]. Deficits in PS, VeLM, and A/V have been found to correlate with NS in subjects with chronic schizophrenia [5,88,143], and to significantly impact functioning through the mediation of social cognition [144].

On the other hand, the results regarding the relationships of NS severity with EF, ToM, Reasoning and Problem Solving, and Verbal Fluency were sparse and inconsistent [31,127,129,130] and could not confirm those observed in the FEP populations (the presence of correlation of NS with EF and ToM, and the absence of correlations with reasoning and problem solving). Interestingly, in relation to EF, a recent meta-analysis by Catalan and colleagues [145] reported that FEP subjects showed a higher intra-group variability when compared to HR subjects, particularly in regard to EF performances, suggesting that the transition to psychosis and the start of treatments may impact neurocognition differently in each individual [146,147,148,149,150].

As briefly discussed, the inconsistency of the retrieved findings can be explained through several factors, including methodological heterogeneity and sample heterogeneity. Indeed, the included studies do show significant methodological heterogeneity, particularly regarding the assessment scales used to evaluate negative symptoms and the underlying conceptual framework. Most studies in first-episode psychosis samples employed assessment scales that are not in line with the NIMH-MATRICS consensus statement on negative symptoms [4]. Regarding this matter, the EPA guidance document on this topic suggests the use of second-generation scales or, when employing first-generation tools, excluding items unrelated to negative symptoms [4]. While only two studies were in line with EPA recommendations [106,109], others employed constructs that included items from various psychopathological domains including cognitive items, such as attention [117,129], difficulty in abstract thinking and stereotyped thinking [68,110,118,119,122], and functioning parameters [125,126,127,128,130,132], that might confound the link between NS and cognitive functions.

Furthermore, in individuals at high risk (HR), most studies utilized assessment scales specifically developed for at-risk populations, notably the SIPS, with three studies also employing the SANS. However, the EPA guidance document and several authors highlighted limitations associated with population-specific tools [4,27,151]. Both SIPS and CAARMS, in fact, include items that reflect outdated conceptualizations of negative symptoms, potentially overlapping with cognitive, affective, and functioning areas. They exhibit validation and psychometric flaws: in particular, the inclusion of cognitive [125,126,127,128,130,132] and functioning items [125,126,127,128,130,132] hinders the analysis of the correlation between negative symptom severity and cognitive impairment, as it may induce an overestimation of the relationship between the two constructs. A minority of studies utilized first-generation scales like the SANS [31,123,129], which the EPA guidance document cautioned against, emphasizing the need for validating versions of second-generation scales tailored to adolescent, high-risk populations.

On the other hand, the current literature shows significant heterogeneity in relation to study samples, with studies differing in relation to both the inclusion/exclusion criteria, and adopted conceptualizations of the FEP and CHR status. As discussed in our introduction and methods sections, in the current review, we attempted to unify the study population to reduce the influence of possible confounding factors, such as differences in the illness phase, antipsychotic treatment, and psychopathological condition. To this end, we decided to focus on early and prodromal stages of illness and to exclude data pertaining to subjects with a diagnosis of affective psychoses. Despite the adoption of narrow criteria, the studies on both FEP and HR subjects included in the present review showed significant discrepancies in relation to the collected samples, which may seriously limit the strength of the reviewed evidence. In relation to first-episode psychosis, the included studies showed significant differences with regards to the illness phase and duration, the duration of antipsychotic treatment, and, significantly, in relation to how FEP patients were defined. Indeed, some studies required patients to be at their first contact with mental health services for psychotic symptoms in order to be defined as FEP, while others set a maximum time distance since the first prescription of antipsychotic medication, thus recruiting, for instance: (1) subjects with less than two [111]; (2) or six weeks [114] of lifetime antipsychotic medication; (3) subjects within their first three months [121]; (4) or first year [109] of ‘adequate’ antipsychotic treatment; (5) subjects within two years since the diagnosis [68]; or, alternatively, (6) antipsychotic-naïve subjects [117].

In relation to at-risk subjects, all the included studies enrolled subjects meeting one of three criteria: a diagnosis of brief limited psychotic symptoms criteria, attenuated positive symptoms and genetic risk, and functional decline, while none of the included studies considered cognitive disturbances as a criterion. On this topic, however, the EPA guidance paper on the early detection of clinical high-risk states of psychoses [152] did not support the use of genetic or functional decline criteria, and recommended the identification of HR states through either attenuated positive, intermittent psychotic, and the presence of cognitive basic symptoms. Additionally, among these three criteria, the attenuated positive symptoms (APS) criterion is the most frequently met [153]; however, the recent literature shows that SIPS and CAARMS exhibit a significant disagreement in the detection of APS, hence potentially leading to the identification and inclusion of hardly comparable subgroups [154]. In addition, recent contributions have shown that the HR samples highly differ in regard to the pre-test risk of psychosis of enrolled individuals (i.e., the probability of included subjects to develop a psychotic disorder before the risk-assessment test is conducted) [155]. This emphasizes the importance of considering recruitment strategies when designing and evaluating a study. Despite this, recruitment strategies in the included studies—as in the current literature in general—were poorly detailed and often highly discrepant.

Taking all these observations into account, data on the relationship between NS and cognitive deficits not only differ, but sometimes diverge within and across non-affective FEP and CHR samples. While these differences may be partially caused by the variability within and between groups in neurocognitive performances [145], they may also suggest that the definitions of FEP and HR do not simply identify populations along a temporal continuum. Consistently with this idea, several studies have highlighted that psychotic-like experiences are also prevalent in subjects with affective and anxiety disorders [156]. Additionally, while the first trials evaluating the conversion rates of CHR individuals reported percentages as high as 40%, the most recent studies indicate that the risk of developing a psychotic disorder for HR subjects may be far lower—between 6 and 20% depending on the trials [157,158,159,160], and that most at-risk individuals will never develop a psychotic disorder [161,162,163]. Moreover, before being identified as FEP, these subjects often exhibit a diagnosis of non-psychotic disorders, such as mood, anxiety, obsessive-compulsive, and eating disorders [164], and receive psychosocial or pharmacological help for non-psychotic conditions before psychotic onset [165,166,167,168]. These findings support the idea that the risk of transition to psychosis is a multidimensional concept, and that HR subjects are a heterogenous group experiencing symptoms pertaining to several psychopathological dimensions [169]. This knowledge is particularly crucial in relation to the topic of the present review, given the well-known influence of both affective and anxiety symptoms on both negative symptoms and cognitive impairment, which may not be psychosis-specific [170,171].

In most of the studies included in the present review, however, included samples were often evaluated in relation to the psychosis risk and symptoms, but under-characterized in relation to other psychopathological areas as well as to environmental factors, probably due to the assumption that only those experiencing subthreshold psychotic symptoms may progress to schizophrenia.

More in general, as summarized in Tables S1 and S2, data on potentially confounding factors for both populations, including pharmacological treatment, educational level, age of onset, duration of untreated illness, and eventual psychiatric co-existing disorders, were missing in many of the retrieved studies. This represents a limitation of our review, as it undermines the evaluation of potential confounding factors, as many of these parameters have been associated with NS severity and cognitive impairment. Pharmacological treatments, for instance, are known to cause secondary negative symptoms [5,8]; as such, more quantitative analyses of their potential influence are needed to improve our knowledge on the link between cognitive deficits and negative symptoms. Although the protocol registration of systematic reviews is still not mandatory, its absence for the present study might represent a further limitation.

In conclusion, a full understanding of the relationships between NS and neurocognitive and social cognition dysfunctions is hindered by the limitations of the current literature. Future studies should be based on shared conceptualizations of first-episode psychosis, at-risk states, as well as on the most recent conceptualizations and evidence on negative symptoms. In addition, they should provide an in-depth characterization of recruited samples, hopefully by using state-of-the-art assessment tools.

## Figures and Tables

**Table 2:** Assessment of NS in FEP subjects.

Applied Assessment Scale		Studies
**PANSS**	**Negative Subscale—total score** N1 Blunted Affect, N2 Emotional Withdrawal, N3 Poor Rapport, N4 Passive/Apathetic Social Withdrawal, N5 Difficulty in abstract thinking, N6 Lack of Spontaneity, and N7 stereotyped thinking	Ayres et al. 2007 [119] Hegde et al. 2013 [68] Lee et al. 2019 [110] Stouten et al. 2017 [118] Saleem et al. 2013 [122] Chan et al. 2006 [105]
	**Negative factor:** N1 Blunted Affect, N2 Emotional Withdrawal, N3 Poor Rapport, N4 Passive/Apathetic Social Withdrawal, N6 Lack of Spontaneity and Flow of Conversation, G7 Motor Retardation, G13 Disturbance of Volition, G15 Preoccupation, and G16 Active Social Avoidance	Huang et al. 2016 [116]
	**Negative factor:** Negative subscale total score plus items G13 Disturbance of Volition and G16 Active Social Avoidance	Piskulic et Addington 2011 [121]
	**Negative factor according to EPA guidance:** N1 Blunted Affect, N2 Emotional Withdrawal, N3 Poor Rapport, N4 Passive/Apathetic Social Withdrawal, N6 Lack of Spontaneity, and Flow of Conversation	Engen et al. 2019 [109]
**SANS**	**Total score**	Rodríguez-Sánchez et al. 2008 [117] Chan et al. 2006 [105]
	**Total score excluding attention scale**	Bliksted et al. 2017 [114] Buck et al. 2020 [104] Ventura et al. 2015 [112]
	**Negative factor according to EPA guidance** excluding attention subscale, inappropriate affect, and poverty in content speech items	Chang et al. 2016 [106]
	**Motivational Deficit:** avolition–apathy and anhedonia–asociality subscales **Expressive Deficit:** Blunted affect subscale and Alogia subscale excluding the poverty of content of speech item	Chang et al. 2017 [107] Chang et al. 2020 [108] Wong et al. 2021 [113]
	**Motivational Deficit:** Avolition–Apathy and Anhedonia–asociality subscales **Expressive Deficit:** Blunted affect subscale and Alogia subscale	Ditlevsen et al. 2020 [115]
	**Individual subscales global scores:** Affective flattening, alogia, avolition, and anhedonia–asociality	Trampush et al. 2015 [111]
**BPRS**	**Negative affect Cluster:** items 16 (Blunted affect), 17 (emotional withdrawal), and 18 (Motor retardation)	Mazza et al. 2012 [120]
**HEN**	**Expressive Deficit:** Affect, Behavior, and speech subscales	Chang et al. 2014 [78]

Summary of the assessment tools used by the included studies for the evaluation of NS in FEP subjects. PANSS: Positive and Negative Syndrome Scale; SANS: Scale for the Assessment of Negative Symptoms; BPRS: Brief Psychiatric Rating Scale; HEN: High Royds Evaluation of Negativity Scale.

**Table 3:** Assessment of NS in HR subjects.

Applied Assessment Scale	Studies
**SIPS:** N1 Social Anhedonia, N2 Avolition, N3 Expression of Emotion, N4 Experience of Emotions and Self, N5 Ideational Richness, and N6 Occupational Functioning	Barbato et al. 2015 [126] Gerritsen et al. 2020 [127] Shin et al. 2016 [125] Lindgren et al. 2010 [128] Meyer et al. 2014 [130] Vargas et al. 2018 [132] Niendam et al. 2006 [124]
**CAARMS**	Pelizza et al. 2021 [131]
**SANS Total score**	Leanza et al. 2018 [129]
**SANS Excluding attention**	Üçok et al. 2021 [31] Glenthøj et al. 2017 [123]

Summary of the assessment tools used by the included studies for the evaluation of NS in HR subjects. SIPS: Structured Interview for Prodromal Syndromes; CAARMS: Comprehensive Assessment of At-Risk Mental States; SANS: Scale for the Assessment of Negative Symptoms.

**Table 4:** Correlations between NS total score and CI domains in FEP subjects.

	Processing Speed	Verbal Fluency	Attention/ Vigilance	Working Memory	Verbal Learning and Memory	Visual Learning and Memory	Executive Functions	Social Cognition
**Significant correlations** **In parenthesis, relevant references**	0.282≤ r ≤−0.353 **3/5 studies:** *-Average*: [116,117] -*Good*: [109]	0.282≤ r ≤−0.353 **2/4 studies:** -*Average*: [118] –*Poor*: [119]	−0.23 ≤ r ≤ −0.34 **4/6 studies:** -*Poor:* [68] -*Average*: [116,118] -*Good:* [105]	−0.191 ≤ r ≤ −2.98 **5/8 studies:** -*Poor*: [68,119] -*Average* [118] -Good [104,110]	r = −0.38 **1/3 studies:** -*Poor:* [68]	r = −0.56 **1/5 studies:** -*Poor:* [122]	−0.25 ≤ r ≤−0.49 **5/8 studies:** -*Poor:* [122] -*Average* [116] -*Good*: [105,106,109]	−0.29 ≤ r ≤ −0.41 **4/6 studies:** *-Average:* [114] -*Poor* [120] -Good [112] -*Poor:* [121]

Range of correlation coefficients, number of studies reporting significant correlations/total number of studies investigating the relationship, and assessed methodological quality for each study reporting a significant correlation between NS total score and cognitive impairment in FEP subjects.

**Table 5:** Correlations between NS total score and CI domains in HR subjects.

	Processing Speed	Verbal Fluency	Attention/ Vigilance	Working Memory	Verbal Learning and Memory	Visual Learning and Memory	Executive Functions	Social Cognition
**Significant correlations** **In parenthesis, relevant references**	−0.21 ≤ r ≤ −0.40 **3/4 studies:** -*Poor*: [128,130] -*Average:* [31]	−0.40 ≤ r ≤ −0.46 **2/3 studies:** -*Average:* [125] -*Poor*: [129]	−0.26 ≤ r ≤−0.32 **3/4 studies:** -*Average:* [31] -*Poor*: [127,130]	−0.21≤ r ≤−0.38 **2/4 studies:** -*Average*: [31] -*Poor*: [127]	=−0.21≤ r ≤−0.38 **4/6 studies:** -*Poor*: [128,130,132] -*Average:* [125]	**0/3 studies**	−0.21≤ r ≤ −0.33 **3/6 studies:** -*Poor*: [130] -*Average:* [31] -*Poor*: [130]	−0.38≤ r ≤ −0.40 **2/3 studies:** -*Poor*: [127,131]

Range of correlation coefficients, number of studies reporting significant correlations/total number of studies investigating the relationship, and assessed methodological quality for each study reporting a significant correlation between NS total score and cognitive impairment in HR subjects.

## Data Availability

Data is contained within the article and Supplementary Materials.

## Supplementary Materials

The following supporting information can be downloaded at: https://www.mdpi.com/article/10.3390/jcm12227095/s1, Table S1: Details of the included studies on First Psychotic Episode; Table S2: Details of the included studies on Subjects at High Risk of Psychosis; Table S3: Neurocognitive and Social Cognition assessment tools adopted in the included studies.

## Abbreviations

A/V = Attention/vigilance; BNSS = Brief Negative Symptom Scale; CAINS = Clinical Assessment Interview for Negative Symptoms; CI = Cognitive impairment; EF = Executive functions; EXP = Expressive Deficit domain; FEP = First-episode psychosis; HR = High risk; MAP = Motivational Deficit domain; NS = Negative Symptoms; PANSS = Positive and Negative Symptoms Scale; SANS = Scale for the Assessment of Negative Symptoms; SC = Social cognition; VeLM = Verbal learning and memory; ViLM = Visual learning and memory.
