# Supplementary material for: Correlations between Negative Symptoms and Cognitive Deficits in Individuals at First Psychotic Episode or at High Risk of Psychosis: A Systematic Review
Source: J Clin Med. 2023 Nov 14;12(22):7095. doi: 10.3390/jcm12227095 (PMC10672428; doi:10.3390/jcm12227095)
Supplement: Supplementary file 1 [file jcm-12-07095-s001.zip › jcm-2599972-supplementary.pdf]

Supplementary materials

Table S1. Details of the included studies on First Psychotic Episode

Author(s), Year, study design	Sample size/ Number of Included Studies and Characteristics of included subjects	Pharmacological treatment	Negative symptom assessment	Cognitive deficit assessment	Results	Study Quality
Bliksted et al. 2017<br><br>Cross-sectional	FES=59<br>Mean Age=22.9y;<br>Gender (n. males)= 36;<br>Mean education =12.1y; Mean age at onset=N/A<br>DUP=N/A<br>days of FES-diagnosis=129.1;<br>DUI=9.5y	SGA=29;<br>SGA(+antidepressant)=13;<br>SGA(+other medications)=4;<br>FGA(+other medication)=1;<br>SGA(+antidepressant+BZD)=1<br>Antipsychotic-naive=9<br><br>Mean CPZe=N/A<br><br>Duration of treatment:<br>patients enrolled between 2009-2010 (n=36): less than 3m of treatment<br>patients enrolled between 2011-2012 (n=23): less than 6w of treatment during lifetime or antipsychotic-naïve	SANS, excluding attention	- Neurocognitive deficits: BACS composite score;<br><br>IQ: WAIS III<br><br>- Social perception: TASIT;<br><br>- Theory of mind: ATT	Blunted affect and alogia correlated negatively with ToM (r -0.30, $p \leq 0.05$, r -0.32, $p \leq 0.01$, respectively), overall social cognition (r=-0.29, $p \leq 0.05$, r=-0.28 , $p \leq 0.05$, respectively) and IQ (r=0.33, p= 0.01, r=-0.44, p=≤0.001, repectively).<br><br>Alogia but not blunted affect correlated negatively with social perception (r -0.32, $p \leq 0.01$)<br>Avolition-apathy correlated negatively with neurocognitive composite score (r -0.30, $p \leq 0.05$);<br>Anhedonia-asociality correlated negatively with social perception, neurocognition, IQ and social cognitive composite score (r=-0.32, $p \leq 0.01$, r=-0.38, $p \leq 0.05$, r=-0.36, p<0.01 r=-0.39, $p \leq 0.01$, respectively).<br><br>Subgroup analysis revealed that patients with high NS had more severe neurocognitive and social cognitive deficits compared to other groups; patients with high positive symptoms but low NS had better neurocognitive and social cognitive performances compared to all other groups.	Average
Buck et al. 2020<br><br>Cross-sectional	FEP= 276 (SCZ=182; SCZ-A=37; DD= 9; BPD=1; Psychosis NOS=47);<br><br>Demographic variables were reported separately	FGA/SGA=N/A<br><br>Mean CPZe:<br>M= 170.23<br>F=148.79<br><br>Duration of treatment: no past antipsychotic medication > 1month	SANS, excluding attention	- Neurocognition: WMS-III or CogState Research Battery depending on recruitment date (before and after 2010 respectively)	The serial mediation model analysis showed male sex was correlated with worse Verbal working memory, which predicted more negative symptoms which predicted worse functioning (β=0.67, SE=0.38, 95% CI=[1.00, 1.57]).	Good

	for male and female subjects:<br><br>Males=201<br>Mean Age=22y;<br>Mean education= 11.73y; Mean age at onset=21.92y.<br>DUP=N/A<br><br>Females= 75<br>Mean age= 23.16y;<br>Mean education=12.25y;<br>Mean age at onset=22.36y<br>DUP=N/A<br>DUI=N/A					
Chan et al. 2006<br><br>Cross-sectional	FES=78<br>Mean age=28.5;<br>Gender (n. males)= 49 M;<br>Mean education= 10.5y<br>Mean age at onset= N/A<br>DUP=248.6d<br>DUI=N/A	Drug-naive	PANSS negative subscale<br>SANS	-Sustained attention: CPT, SART, MCT<br><br>-executive functions: LNS, MWCST, VPT, TMT-B, HSCT-B, SET, Stroop test<br><br>-Reasoning and problem solving: Tower of Hanoi Test	Initiation components were found to be associated with PANSS negative symptom (r = 0.34, p = 0.0004) and SANS global alogia (r = 0.3, p = 0.01), but also with memory impairment and global IQ. Sustained attention component was also significantly correlated with PANSS negative symptoms (r =-0.34, p = 0.003), and SANS global alogia (r =-0.29,p = 0.01) but also general psychopathology and IQ. Online updating component was associated with PANSS negative symptoms (r =-0.27, p = 0.02) but also with memory impairment and IQ. Switching and flexibility component was related to PANSS negative symptoms (r = 0.25, p = 0.04) and SANS global affective flattening (r = 0.25, p = 0.04) but also with memory impairment and IQ.	Good
Chang et al. 2014<br><br>Longitudinal and cross-sectional	FEP-SSD=93 (SCZ=75; Schizophreniform=13 SCZ-A=5);<br>Mean age=31.2;<br>Gender (n. males)= 42 M;<br>Mean education= 10.4y<br>Mean age at onset= N/A	N/A	High Royds Evaluation of Negativity Scale (HEN): EXP score comprises Affect, Behavior and Speech subscales	-Logical memory test: WMS-R<br><br>-Visual learning and memory: WMS-R<br><br>- Processing speed: DSF<br><br>- Executive functions: MWCST	EXP was found to correlate with cognitive deficits and particularly with EF concurrently at different time points but not in longitudinal analyses.<br><br><u>Cross-sectional relationships:</u><br>EXP was significantly associated with verbal fluency at 12 months and 24 months (r=−0.32, p <.0083, r=−0.33, p <.0083 respectively), but not at 36 months.<br>EXP was significantly associated with visual learning and memory at 24 and 36 months (r=−0.34,	Average

	DUP=473d<br>DUI=N/A			-Category verbal fluency	p < 0.0083, r=−0.40, p<0.0083) but not at 12 months and executive functions;<br>EXP was significantly related to executive functions at 24 and 36 months (r=−0.36, p ≤0.05, r=−0.29, p < 0.0083) but not at 12 months.<br><br><u>Longitudinal relationships:</u><br>No cross-lagged associations between EXP and cognition were found. No significant correlations between changes in EXP severity and cognitive impairment were observed over three years	
Chang et al. 2016<br><br>Cross-sectional	FEP = 355<br>(SSD = 36;<br>BPD=4; DD=10;<br>Psychosis NOS=2):<br><br>FEP with Primary NS (FEP-PNS, score >3 in at least one of the four SANS subscales but excluding depression, Parkinsonism and positive symptoms) = 52<br>Mean Age=38y;<br>Gender (n. males)= 14;<br>Mean education =10.29y; Mean age at onset=36.2y<br>Log DUP =1.95<br>DUI=N/A<br><br>FEP without Primary NS (FEP-noPNS) = 303<br>Mean Age=38.3y;<br>Gender (n. males)= 127;<br>Mean education =10.7y; Mean age at onset=36.6<br>Log DUP=1.95<br>DUI=N/A	SGA:<br>FEP-PNS =38<br>FEP-noPNS =217<br><br>Mean CPZe:<br>FEP-PNS = 190.42<br>FEP_noPNS = 168.14<br><br>Mean duration of treatment: 119.7d	SANS, excluding attention subscale, “inappropriate affect” and “poverty of content of speech” items	- Working memory: DSF and DSC of WAIS-R;<br><br>- Visual Learning and Memory: WMS-R Logical memory and visual reproduction subtests;<br><br>- Executive functions: MWCST	There was a significant between-group difference in cognitive functions:<br>FEP-PNS exhibited poorer working memory (f= 6.62, p≤0.01) and executive functions than FEP_noPNS (f= 6.62, p≤0.01);<br>no differences were found between PNS and no PNS in logical memory and visual reproduction performance.<br><br>Premorbid social functioning and working memory were independently associated with PNS status.	Good

Chang et al. 2017<br><br>Longitudinal	FEP = 321<br>CB-SCID-I/P (DSM IV) and IRAOS (SSD=201; Other non-affective psychoses=120);<br>Mean Age=38.3y;<br>Gender (n. males)= 141;<br>Mean education =10.8y; Mean age at onset=36.5y;<br>DUP=531.7d<br>DUI=N/A	FGA/SGA=N/A<br><br>Mean duration of treatment=119.7d<br><br>Mean CPZe=N/A<br><br>Mean duration of treatment=N/A	SANS negative domains: Motivational Deficit was composed by the Avolition-apathy and Anhedonia-asociality subscales; Expressive Deficit was composed by Blunted Affect subscale and the poverty of speech item of the Alogia subscale	- Working memory:WAIS-R DSF/DSB and DSC;<br><br>-Executive Functions: MWCST	Working memory, executive functions and verbal fluency did not correlate with Expressive Deficit and Motivational Deficit as evaluated with the SANS<br><br>The relationship between neurocognition and functioning was partially mediated by amotivation (indirect effect: β=0.11, p< 0.001).	Good
Chang et al. 2020a<br><br>Network analysis	FEP= 323 CB-SCID-I/P (DSM-IV) :<br>(SCZ = 144; SCZ-phreniform= 58; SCZ-A= 3; BPD= 40 DD= 60 ;PD-nos= 18)<br>Mean age= 38y;<br>Gender (n. males)= 140; mean education= 10.9y<br>Mean age at onset=36.3<br>DUP= 536.7d<br>DUI=N/A	FGA/SGA=N/A<br><br>SGA medication= 229<br><br>Mean CPZe = 168.5<br><br>Mean duration of treatment=N/A	SANS negative domains: Motivational Deficit was composed by the Avolition-apathy and Anhedonia-asociality subscales; Expressive Deficit was composed by Blunted Affect subscale and the poverty of speech item of the Alogia subscale	-Working memory: WAIS-R DSF/DSB<br>- Processing speed: WAIS-R DSC<br><br>- verbal fluency: MWCST	MAP played the most central role and had the strongest associations with other nodes in the constructed network, especially with the role functioning but not with cognition; only PANSS disorganization had a correlation with processing speed.	Good
Ayres et al. 2007<br><br>Cross-sectional	FEP=179 (SCZ/SCZphreniform disorder=98)<br>mean age=32.2y;<br>Gender (n. males)= 86;<br>Education: less than 5y=22.9%; 5-8y=24.7%; 9-	FGA/SGA=N/A<br><br>FGA=97;<br>SGA=27<br><br>Anticholinergic drugs=50<br><br>Mean CPZe=N/A	PANSS Negative subscale	Verbal fluency: COWAT;<br>- Verbal working memory: DSF, DSB	Negative symptoms significantly correlated with verbal fluency (r=0.402, p<0.001), DSF, (r=0.191, p<0.001) and DSB (r=0.282, p<0.001) performances.	Poor

	11y=32.9%; 12y or more=19.4%;<br>Mean age at onset=N/A<br>DUP=264.7d<br>DUI=2y	Mean duration of treatment=97.8d				
Ditlevsen, Simonsen, and Bliksted 2020<br><br>Cross-sectional	FES=89<br>mean age=23.6y;<br>Gender (n. males)= 35;<br>mean education=12.9y<br>Mean age at onset=N/A<br>DUP=N/A<br>DUI=N/A	FGA/SGA=N/A<br><br>Mean duration of treatment=215d	SANS total score (excluding attention), Motivational Deficit and Expressive Deficit	- Theory of mind: ATT	ED symptom severity was the best predictor of TOM performance (AIC weight = 0.44) compared to AA symptom severity, AA+DE combined and total SANS score; however, ED only explained 1-2% of the variance in ATT performance.	Average
Engen et al. 2019<br><br>Longitudinal study	FEP-SSD = 87<br>4 groups based on NS severity:<br><br>Sustained NS= 26<br>mean age=26.3y;<br>Gender (n. males)= 22 M; mean education=12.5y;<br>age at onset=23.1y;<br>DUP (Log)=1.7;<br>DUI=N/A<br><br>Transient NS= 23:<br>mean age=26.9y;<br>Gender (n. males)= 8 M; mean education= 12.3y;<br>age at onset=21.9;<br>DUP(Log)=1.9<br>DUI=N/A<br><br>Mild NS= 26<br>mean age=28.1y;<br>Gender (n. males)= 16 M; mean	FGA/SGA=N/A<br><br>Antipsychotic medication (DDD=Defined Daily Dosage):<br>Ssustained NS=1.1<br>Transient NS=0.9<br>Mild NS=0.9<br>No NS=0.7<br><br>Mean CPZe=N/A<br><br>FEP were recruited within the first 52 weeks after start of first adequate treatment (antipsychotic medication/hospitalization)	PANSS negative symptoms as defined by EPA Guidance:<br>N1 (affective flattening), N2 (emotional withdrawal), N3 (poor rapport), N4 (passive/apathetic social withdrawal), N6 (lack of spontaneity and flow of conversation	IQ: WASI<br><br>-Verbal Learning and Memory: CVLT-II (verbal learning and recall); WMS (Logical Memory, immediate and delayed recall)<br><br>-Processing Speed: WAIS- III (Digit Symbol Test) and D-KEFS (Color-Word Interference Test);<br><br>-Attention: Digit Span and Letter Number Sequencing Test;<br><br>-Executive Functions: D-KEFS (Inhibition and Inhibition/Switching subtest from the Color-Word Interference Test;	NS had a significant and large overall effect on cognitive performance in the 4 cognitive domains;<br><br>All NS groups except the NNS performed significantly poorer than HCs on all domains, except MNS for verbal learning and memory;<br><br>The SNS group was outperformed by the NNS group on processing speed and executive functions, and on verbal learning and memory by the MNS and TNS.<br><br>No difference in cognitive course between the NS groups over the 1-year follow-up period was found.<br><br>The cognitive composite score was strongly and negatively correlated with the total level of NS both at baseline (r=−0.36, p=.001) and follow up (r= −0.32, p=.003)	Good

	education= 12.4y;<br>age at onset=24.7y;<br>DUP(Log)=1.5;<br>DUI=N/A<br><br>No NS= 12<br>mean age=27y; 6 M;<br>mean education= 11.8y;<br>age at onset=24y;<br>DUP(Log)=1.6<br>DUI=N/A			- Verbal Fluency: Letter Fluency, Category Fluency and Category Switching from the Verbal Fluency test		
Hegde et al. 2013<br><br>Cross-sectional	FES=49 (Paranoid SCZ=44; Undifferentiated SCZ=5);<br>mean age=29.18;<br>Gender (n. males)= 34;<br>mean education=11.9y;<br>Mean age at onset=N/A<br>DUP=N/A<br>DUI=12±8m	antipsychotic medication=100% (FGA/SGA=N/A)<br>Mean CPZe=N/A<br><br>Mean duration of treatment=N/A	PANSS negative symptoms subscale	-Sustained attention: Digit Vigilance Test;<br>-Focused attention: Color Trails Test;<br>-Divided attention: Triads test;<br>-processing speed: DSST;<br>-verbal category fluency: Animal names test<br>-verbal working memory: Verbal N-back-task<br><br>- Executive functions: planning: Tower of London,<br>-concept formation and set shifting ability: WCST<br>-verbal learning and memory: RAVLT;<br>-Visual learning and memory: ROCFT	Negative symptoms scores correlated significantly with sustained attention (r 0.31, p <0.05), verbal working memory (r=-0.29, p <0.05), planning (r=0.29, p<0.05), concept formation and set shifting ability (r=-0.29, p<0.05) and verbal learning and memory (r=-0.38, p<0.01).<br><br>Negative symptoms did not correlate with focused attention, divided attention, processing speed, verbal fluency, and visual learning and memory.	Poor
Huang et al. 2016<br><br>Cross-sectional	FES=92<br>mean age=22.8y;<br>Gender (n. males)= 36;<br>mean education=10.7y	drug-naive	PANSS negative factor<br><br>N1 Affective flattening, N2 Emotional Withdrawal, N3 Poor Rapport,	- Executive functions: WCST<br><br>- Cognitive flexibility: SCWT	The negative factor scores were negatively correlated with EF tasks scores (r= 0.272 - 0.381, p <0.01), A/V scores (r= 0.230, p <0.05), processing speed (r= 0.293 – 0.306, p <0.01), and cognitive flexibility (r= 0.331 – 0.409, p <0.01),	Average

	Mean age at onset=N/A<br>DUP=N/A<br>DUI=12.2m;		N4 Passive/Apathetic Social Withdrawal, N6 Lack of Spontaneity and Flow of Conversation, G7 Motor Retardation, G13 Disturbance of Volition, Preoccupation, and G16 Active Social Avoidance	- Attention/Vigilance: CPT;<br><br>- Processing Speed, executive functioning: TMT		
Lee et al. 2019<br><br>Longitudinal	FEP = 160: (SCZ=80;DD=19; BPD=33; SCZ-A=1 Psychosis nos=27);<br>Mean Age=31.2y;<br>Gender (n. males)=63;<br>mean education= 11.6y;<br>Mean age at onset=N/A<br>DUP=90d;<br>DUI=N/A<br>62.5 % inpatients	SGA/FGA=N/A<br><br>CPZe = 338.8 mg/day<br><br>within 1 month of antipsychotic treatment	PANSS negative subscale	-Working Memory: DSF/DSB, LNS<br><br>-Processing speed: DSC<br><br>-Visual learning and memory: WAIS-R visual patterns test	NS, general psychopathology and insight were significantly associated with cognition and were predictive of functioning;<br>NS, general psychopathology and insight were significant mediators between cognition and functioning.<br><br>The significant direct relationship between cognition and functioning became insignificant after including symptoms and insight in the model. PANSS negative scores significantly correlated with logical memory (r=−0.301, p<0.01), working memory DSF/DSB (r=−0.233, p<0.05) and LNS scores (r=−0.296, p<0.01) but not with DSC and visual learning and memory performance (r=−0.161 −0.202).	Good
Mazza et al. 2012<br><br>cross-sectional	FEP= 49 (SSD=49; within 3 months from initial diagnosis);<br>Mean age= 26.4;<br>Gender (n. males)= 33;<br>Mean education= 12.6y<br>Mean age at onset=N/A<br>DUP=N/A<br>DUI=N/A	N/A	BPRS: item 16, (Affective flattening), 17 (emotional withdrawal), 18 (Motor retardation)	- TOM= TOM advanced task	Negative symptoms were significantly and negatively correlated with TOM scores (r=-,383, p<0.000).	Poor

Piskulic and Addington 2011<br><br>Report study	FEP=50 (SCZ=32; SCZ-phreniform=12; DD=1; Psychotic disorder NOS=3; SCZ-A=1);<br>mean age=25.1y;<br>Gender (n. males)= 15 M<br>Mean education= N/A<br>Mean age at onset=N/A<br>DUP=N/A<br>DUI=N/A	N/A	PANSS negative symptoms subscale plus Disturbance of volition (G13) and active social avoidance (G16)	-Facial affect recognition: FEIT, FEDT<br>-Social perception: SCRT<br>-Social knowledge: SFRT	Stereotyped thinking (N7) was the only item that made a significant unique contribution to each of the three meausures of social cognition;<br>N1, N6, N7 and G16 significantly predicted 20% of the variance in facial affect recognition;<br>N5, N6, N7 explained 33% of the variance in social knowledge;<br>N3, N4, N5, N6, N7 explained 25% of the variance in social perception	Poor
Rodríguez-Sánchez et al. 2008<br><br>Cross-sectional	FEP=126<br>no prior treatment with antipsychotic medication or, if previously treated, a total lifetime of adequate antipsychotic treatment of less than 6 weeks.<br><br>(SCZ=77; SCZphreniform=34; BPD=8;<br>Psychosis NOS=7);<br>Mean age=26.9y;<br>Gender (n. males)= 81;<br>mean education=10.32y;<br>Mean age at onset=N/A<br>DUI=24.6m;<br>DUP=12.13m;<br>Inpatients=82	FGA=42<br>SGA=84<br><br>Mean CPZe=N/A<br><br>Mean duration of treatment=no prior treatment or, if previously treated, a total life time of adequate antipsychotic treatment of less than 6 weeks	SANS total score	-Attention/ Vigilance: CPT;<br><br>-Verbal learning and memory: RAVLT;<br><br>-Visual learning and memory: Rey complex figure;<br><br>-Executive functions and speed of processing: Visual cancellation test, DSF/DSB, TMT-A, TMT-B<br><br>- Verbal fluency: FAS	TMT-A (r=-0.287, p=0.002)/B (r=-.227; p=0.014), visual cancellation (-0.391; p<0.001) and digit symbol (r=-0.202, p=0.029), correlated with negative symptoms.<br><br>No correlation was found between NS with attention, verbal and visual memory, verbal fluency nor verbal ability.	Average
Saleem et al. 2013	FEP= 20 (max 1 episode, diagnosis NOS)<br>Mean age=26.5y;<br>Gender (n. males)=	SGA =17<br>FGA=3<br><br>Mean CPZe=N/A	PANSS negative subscale	CANTAB<br>-Visual learning and memory: PRM;	Visual learning and memory (r = -0.56, p < 0.01) and executive functions (r = -0.49, p < 0.05) were significantly and negatively correlated with the severity of negative symptoms. However, there were	Poor

Cross-sectional study	16;<br>mean education=11.9y<br>Mean age at onset=N/A<br>DUP=N/A<br>DUI=N/A	duration of treatment=N/A		-Visual learning and memory: SRM;<br><br>-Executive functions: IED;<br><br>-Working memory: SOC;<br><br>-Attention: CRT	no significant correlations with working memory or attention.	
Stouten et al. 2017<br><br>cross-sectional study	FEP=162 (enrollment within the first three months from first contact) (SCZ=81<br>SCZ-aff=9<br>BPD=9<br>DD=5<br>shared psychotic disorder=2<br>psychotic disorder NOS=56)<br>Mean age=27.61y;<br>Gender (n. males)= 116;<br>mean education=11.9y<br>Mean age at onset=N/A<br>DUP=N/A<br>DUI=N/A	N/A	PANSS negative subscale	-Cognitive biases: DACOBS<br><br>-Emotion processing: ANT<br>-Theory of mind: Hinting task<br>-Social knowledge: WAIS-III picture arrangement<br><br>- Processing speed: TMT-A<br><br>- Working memory: WAIS III LNS<br>- Verbal learning and memory: RAVLT<br><br>- Attention: CPT<br><br>- Verbal fluency: Category fluency task<br><br>- Problem solving: Tower of London, WAIS III block design subset<br><br>- Working memory: WAIS III LNS<br><br>- Visual learning:	Negative symptoms were significantly correlated with 'verbal processing speed', a factor composed of processing speed, attention, verbal learning and memory and verbal fluency tests ($r=-0.353$, $P \leq 0.001$.) but not with a 'general neurocognition' factor (problem solving, working memory, planning), nor social cognition scores.	Average

				BVMT		
Trampush et al. 2015<br><br>Longitudinal study	FES =175<br><br>(SCZ = 123; SCZ-phreniform = 35; SCZ-Aff=5; PD-NOS=12);<br>mean age=22.5y;<br>Gender (n. males) = 128<br>mean education=N/A.<br>Mean age at onset=N/A<br>DUP=N/A<br>DUI=N/A	SGA=175<br><br>93 = randomized to aripiprazole<br>82 = randomized to risperidone<br><br>Mean CPZe=N/A<br><br>Duration of treatment:<br>all participants had less than 2w of prior antipsychotic exposure;<br>49 were antipsychotic-naive	SANS (excluding attention subscale)	MCCB:<br>-Processing speed: SCCT and TMT-A<br>-Working memory: LNS and Spatial span<br>-Reasoning and Problem Solving: NAB Mazes<br>-Verbal Learning and Memory: HVLT-R<br>-Visual Learning and Memory: BVMT-R<br>-Attention/vigilance: CPT-IP<br>-Emotional processing: MSCEIT<br>-Global cognitive performance: Overall composite score	Improvements in general cognitive functioning after 3 m months were fully mediated by improvements in alogia (β = -1.53 p= 0.0005):<br><br>Improvements in working memory were partially mediated by reductions in alogia (β = -2.00 p= 0.0057);<br><br>Improvements in verbal learning were fully mediated by reductions in alogia (β = -1.65 p=0.0007)	Good
Ventura et al. 2015<br><br>cross-sectional and longitudinal	Recent-onset (<2y)<br>SSD=77<br>(SCZ=50; SCZ-A=7; SCZphreniform=20);<br>mean age=21.4y;<br>Gender (n. f males)= 60 M;<br>mean education=12.3y;<br>Mean age at onset=N/A<br>DUP=N/A<br>DUI=N/A<br>illness duration=7.1m	SGA=77<br><br>Mean CPZe=N/A<br><br>All patients were on a stable out-patient dose of risperidone for at least 3w	SANS (excluding attention subscale)	- Neurocognitive composite score: MCCB<br><br>- ToM appropriateness/ intentionality/ length: SAT	At baseline and after 6-month follow-up, both ToM intentionality and appropriateness were significantly related to negative symptoms (r=−0.29, p < 0.01; r= −0.24, p < 0.05 at baseline and r= −0.41, p < 0.01, r= −0.50, p < 0.01 at follow-up).<br><br>TOM impairment was found to correlated with both MAP and EXP both at baseline and follow-up.	Good
Wong et al. 2021<br><br>Cross-sectional	FEP=347 (SSD= 217, psychotic disorder NOS= 130);<br>mean age= 38.3;<br>Gender (n. males)= 151;	FGA/SGA= N/A<br><br>Mean CPZe= 170.6 mg<br><br>Mean duration of treatment= N/A	SANS<br>MAP: items of the Avolition-apathy and Anhedonia-asociality	- Executive functions, verbal fluency: MWCST;<br><br>- Processing speed: DSC;	MAP was significantly correlated with processing speed category verbal fluency and executive function scores (r=-0.235, p < 0.001, r= -0.145, p < 0.01, r=-0.171, p < 0.01 respectively)<br><br>EXP was significantly correlated with processing speed and verbal fluency (r=.201, p < 0.001, r= .190	Good

	mean education=10.7y; Mean age at onset=N/A DUP=17.4y DUI=N/A		subscales of SANS; ED: items of the Affective flattening subscale and the poverty of speech item of the Alogia subscale	Working memory: digit span subtest;<br><br>- Verbal fluency: Category verbal fluency	$p < 0.001$ respectively)	

ANT= Amsterdam Neuropsychological Task ;**ATT**= Animated triangles task**; BPD=** Brief Psychotic Disorder; **BVMT**= Brief Visuospatial Memory Task; **CPT**=Continuous Performance Test; **CRT**=Choice Reaction Time; **CVLT**=California Verbal Learning Test; **DACOBS**= Davos Assessment of Cognitive Biases Scale; **DD** = Delusional disorder; **D-KEFS=** Delis–Kaplan Executive Functioning System; **DSB**=Digit Span Backword; **DSF**= Digit span forward; **DSST**=Digit Symbol Sostitution Test; **DSC**= Digital Symbol Coding subtest **DUP**=Duration of Untreated Psychosis; **EXP**= Expressive Deficit domain of Negative Symptoms; **FEDT**= Face Emotion Discrimination Task; **FEIT**= Face Emotion Identification Test; **FEP** = First-Episode Psychosis; **FES**=First-Episode Schizophrenia; **HC**= healthy control; **HEN**= High Royds Evaluation of Negativity Scale; **HSCT-B**= Hayling Sentence Completion Test Part B **HVLT**= Hopkins Verbal Learning Test; **HVLT-R**= Hopkins Verbal Learning Test – Revised Version; **IRAOS**=Interview for Retrospective Assessment of Onset of Schizophrenia; **LNS**=Letter-Number Span; **MAP**= Motivational Deficit Domain of Negative Symptoms; **MCCB** = Matrics Consensus Cognitive Battery; **MCT**= Monotone counting test; **MSCEIT**= Mayer-Salovey-Caruso Emotional Intelligence Test; **MWCST**= Modified Wisconsin Card Sorting Test; **NAB**= Neuropsychological Assessment Battery **NS**= Negative Symptoms; **PD- NOS**=Psychotic disorder, not otherwise specified; **PNS**= Primary Negative Symptoms; **PRM**= Pattern Recognition Memory; **RAVLT**=Rey Auditory Verbal Learning Test; **ROCF**= Rey Osterrieth complex figure test; **SART**= sustained attention to response task **; SAT**= Social Animation Task; **SANS**= Scale for the Assessment of Negative Symptoms; **SCID**=Structured Clinical Interview; **SCRT**= Social Cue Recognition Test **SCWT**=Stroop Color and Word Test; **SCZ**= Schizophrenia disorder; **SCZ-Aff=** Schizoaffective disorder; **SCZ-f**= schizophreniform disorder; **SFRT**= Situational Features Recognition Test**; SET**= Six element test; SGA=Second Generation Antipsychotic; **SRM**= Spatial Recognition Memory; **SSD**= Schizophrenia Spectrum Disorder; **TASIT**= The Awareness of Social Inference Test; **TMT**=Trail Making Test; **WAIS-III**=Wechsler Adult Intelligence Scale – Third Edition; **WAIS-R=** Wechsler Adult Intelligence Scale – Revised; **WASI**= Wechsler Abbreviated Scale of Intelligence; **WCST**= Wisconsin Card Sorting Test; **WMS-III**= Wechsler Memory Scale—Third Edition; **WMS-R**=Wechsler Memory Scale-Revised.

Table S2. Details of the included studies on Subjects at High Risk of Psychosis

Author(s), Year, study design	Sample size/ Number of Included Studies and Characteristics of included subjects	Pharmacological treatment	Negative symptom assessment	Cognitive deficit assessment	Results	Study Quality
Barbato et al. 2015	CHR=675(SIPS) Mean Age=18.5y; Gender (n. males)= 389; Mean education =11.28y	N/A	SOPS (N1-N6)	-Theory of mind: TASIT -Facial affect processing: ER40 and EDF40 -Social perception: RAD	There were no significant correlations between any of the social cognition measures and NS.	Poor

Cross-sectional						
Gerritsen et al. 2020<br><br>Cross-sectional	CHR = 91 (recruited with COPS, confirmed with the SIPS);<br>mean age=20.8;<br>Gender (n. males)=57<br>Mean education= N/A	N/A	SOPS (N1=social anhedonia or withdrawal;<br>N2=avolition;<br>N3=decreased expression of emotions;<br>N4=decreased experience of emotions and self;<br>N5=impoverished thinking;<br>N6=deterioration in role functioning)	MCCB<br><br>Speed of processing: Symbol-Coding: TMTA<br><br>Verbal Fluency: Animal Naming<br><br>Attention/vigilance: CPT<br><br>Working Memory: LNS<br><br>Verbal learning and memory: HVLT-R<br><br>Visual learning and memory: BVMT<br><br>Reasoning and problem solving: Nab Mazes<br><br>Social cognition: MSCEIT	Canonical correlation revealed a single canonical correlation between cognition and NS which accounted for 38% of the variance in each canonical variate.<br>A single cognitive factor composed of verbal working memory, social cognition and vigilance is associated with NS;<br>LNS scores had the highest loading onto the canonical variate, indicating a high degree of overlap between verbal working memory and negative symptoms. The canonical variate accounted for 86% of the variability in LNS scores.	Poor
Glenthøj et al. 2017<br><br>Cross-sectional	UHR=84<br>(CAARMS);<br><br>(Affective disorder=48;<br>anxiety disorder=50;<br>substance use disorder=9;<br>somatoform disorder=3;<br>eating disorder=2;<br>adjustment disorder=2;<br>personality disorder=15);<br>mean age=24.4y;<br>Gender (n. males)=35;<br>mean education=14.3y	38 were taking antipsychotic treatment	SANS<br>(excluding attention subscale)	BACS composite score	NS significantly mediated the relationship between neurocognition and functioning: PSP (β = 0.23, p = 0.02), GF:Social (β = 0.23, p = 0.02), GF:Role (β = 0.23, p = 0.02), and SOFAS (β = 0.23, p = 0.02).	Good
Lindgren et al. 2010b<br><br>Cross-sectional	CHR=62<br>(SIPS)<br><br>mean age=16.5;<br>Gender (n. males)=43 M;<br>students in eight-	N/A	SIPS (N1=social anhedonia or withdrawal; N2= avolition;<br>N3=decreased expression of emotions;	- Processing speed: TMT-A, TMT-B, TMT-C, DSC<br><br>- Verbal performance: verbal learning, vocabulary, visual reproduction	Negative symptoms correlated negatively with processing speed (r=-0.31; p=.014) and verbal performance (r=-.37; p=.003); no correlations were found with visuospatial performance.	Poor

	grade/senior high school=0; inpatients=18		N4=decreased experience of emotions and self; N5=decreased ideational richness (comprehension and abstraction); N6=deterioration in role functioning)	- Visuospatial performance: visual reproduction, block design, matrix reasoning, similarities		
Leanza et al. 2018b<br><br>Cross-sectional	CHR = 154 (BSIP);<br>mean age=25.9y;<br>Gender (n. males)= 104;<br>mean education=11.6y	Antipsychotics currently:<br>Untreated=142<br><br>SGA=11<br><br>(total cumulative lifetime dose = 2500 CPZ equivalents)	SANS total score and subscales	Verbal IQ: MWT-A;<br><br>Non-Verbal IQ: LPS;<br><br>Planning Ability: Tower of Hanoi;<br><br>Cognitive Flexibility: WCST;<br><br>Verbal Fluency: VF Test;<br><br>Verbal Learning and memory: CVLT;<br><br>Selective Attention and Reaction Inhibition: TAP Go/No-Go;<br><br>Working memory: TAP-WM;<br><br>Vigilance: CPT	SANS total score was significantly and negatively correlated with verbal fluency (r =-0.040, p = 0.020) and non-verbal IQ (r = -0.020, p = 0.040), but not with Planning, Cognitive flexibility, Verbal learning & memory, Selective attention & inhibition, Working memory, or Vigilance.<br>Alogia was significantly associated with both nonverbal IQ (r = 0.024, p < .05) and verbal fluency (r = 0.024, p < .05).<br>Asociality/Anhedonia was significantly associated with nonverbal IQ (r = .023, p < .05).<br>The subscales Affective Flattening, Avolition/Apathy and Inattention did not withstand correction for multiple testing.	Poor
Meyer et al. 2014<br><br>Longitudinal	CHR: 371 (SIPS);<br>mean age=18.2;<br>Gender (n. male)= 234<br>Mean education= N/A	6 patients were taking antipsychotic medications (3.6%)	SIPS (social anhedonia or withdrawal, avolition, decreased expression of emotions, decreased experience of emotions and self, decreased ideational richness, deterioration in role functioning)	-Verbal memory: Vocabulary;<br><br>-Visual-perceptual-organization:Block Design;<br><br>Attention/Vigilance: CPT, identical pairs version;<br><br>-Processing speed:DSC;<br><br>-Verbal fluency: COWA;<br>- problem solving: WCST	SIPS-negative score correlated with verbal comprehension (r=−0.21 p <0 .01), processing speed measured with both digit symbol and TMT-A (r=−0.34 p < 0.001, r=−0.21 p< 0.01), vigilance (r=−0.32 p < 0.001), verbal fluency (r=−0.24 p < 0.01), problem solving (r=−0.33 p < 0.001) and verbal memory (r=-0.30 p < 0.001).<br><br>SIPS-social anhedonia correlated with lower problem-solving scores (r=-0.20, p < 0.05), processing speed (r=-0.22, p < 0.05) and global neurocognition (r=-0.23, p < 0.05),	Poor

					but not with vocabulary, visual perceptual organization, attention/vigilance;<br>SIPS-avolition correlated with lower processing speed (r = -0.20, p < 0.05), problem solving (r = -0.19, p < 0.05) and global scores (r=-0.20, p < 0.05) and vigilance, but not with vocabulary, organization, fluency. and vigilance;<br>SIPS-Decreased expression of emotions correlated with verbal fluency (r=-0.18, p <0 .05), problem solving (r=-0.19, p < 0.05), processing speed (r=-0.20, p < 0.05), global neurocognitive scores (r= -0.22, p < 0.05), but not with vocabulary, organization and vigilance;<br>SIPS-Decreased experience of emotions did not correlate with neurocognition;<br>SIPS-decreased ideational richness correlated with each neurocognitive test score (r= -0.18 to -.36, p < 0.05);	
Niendam et al. 2006<br><br>Cross-sectional	CHR: 45 (SIPS);<br>mean age=17.66;<br>Gender (n. male)= 29<br>Mean education= N/A	SGA= 19<br>mood stabilizers=6<br>antidepressants=22<br>psychostimulants=3	SIPS (social anhedonia or withdrawal, avolition, decreased expression of emotions, decreased experience of emotions and self, decreased ideational richness, deterioration in role functioning)	-Processing speed: TMT-A/B, DSC, FAS and Animal Naming subtests of the Verbal Fluency Test<br><br>-Reasoning and problem solving: WASI Matrix Reasoning<br><br>-Visual learning and memory: WMS-III Visual Reproduction Immediate and Delayed Recall<br><br>-Verbal learning and memory: CLVT<br><br>Verbal working memory: Digit Span Backwards	SOPS Negative Symptom score was not significantly correlated with any neurocognitive variables of interes	Average
Pelizza et al. 2021	UHR = 51 (CAARMS early version)<br>mean age=15.5y;	Low-dose atypical antipsichotic were	CAARMS factor 1 ("Negative-	Subjective experience related to cognitive: I-GEOPTE		Poor

Longitudinal	Gender (n. males)=20;<br>mean education=10.3;<br>DUI=61w	used only in subjects who:<br>–had an imminent risk of suicide or severe violence or<br>-were overwhelmed by abruptly worsening overt psychotic symptoms<br>or<br>-were rapidly deteriorating in daily functioning<br>or<br>-did not respond to any other treatment	interpersonal Dimension”)	“Basic cognitive functions” subscore<br><br>Subjective experience related to social cognitive impairment: I-GEOPTE “Social Cognition” subscore	The GEOPTE “Basic Cognitive Functions” subscale score did not show any correlation with CAARMS factor 1 score;<br><br>The GEOPTE “Social Cognition” subscale score showed significant positive correlations with CAARMS factor 1 score (r = 0.400, p= 0.0001);<br><br>After 2 years of follow-up, ∆ T0-T2 I-GEOPTE total score showed significant positive correlations with ∆ T0-T2 CAARMS Negative symptoms score (r=0.747, p= 0.0001), Avolition-apathy in particular (r=0.803, *p* = 0.0001)	
Shin et al. 2016<br><br>Longitudinal	CHR=47<br>(CAARMS early version+SIPS);<br>mean age=19.3y;<br>Gender (n. males)= 33;<br>mean education=12.0y	N/A	SOPS-negative	- Working memory: digit test from K-WAIS<br>-Processing speed: TMT-A<br>-Executive function: TMT-B; WCST- Perseveration<br>-Verbal fluency: letter and semantic fluency<br>-Verbal memory: K-CVLT<br>-Visual memory: RCFT	After the 2-year follow-up, the change of semantic fluency significantly related to changes in SOPS-negative (r=0.460, p=0.001);	Average
Üçok et al. 2021<br><br>Cross-sectional	UHR = 107<br>(Three criteria: (a) BLIPS, (b) attenuated psychotic symptoms and (c) family risk with reduced function)<br>mean age=20.4y;<br>Gender (n. males)= 83<br>mean education=10.5y	Antipsychotic naïve	SANS (excluding attention subscale)	-Verbal learning and memory: RAVLT;<br><br>-Selective attention, interference inhibition, processing speed, cognitive flexibility and executive functions: SCWT<br><br>-Executive function and working memory: WCST<br><br>-Working memory: DSB;<br><br>-Processing speed, sequencing, mental flexibility and working memory: TMT A/B	TMT-A (time) was positively correlated with SANS-alogia (r=0.26, p<0.05); TMT-B was positively correlated with SANS total score (r=0.40; p<.001) and with all the SANS subdomain scores (affective: r= 0.35; p<0.001; alogia: r= 0.42; p<0.001; avolition: r= 0.29; p <0.01; anhedonia: r= 0.30, p<0.01).<br>Executive functions and working memory were negatively correlated with SANS total (r=-0.21, p<0.05), SANS-affective (r=-0.20, p<0.05) and SANS-alogia (r=-0.23, p<0.05).<br>Attention composite score was negatively correlated with SANS total score (r=-0.26, p<0.01), SANS-affective (r=-0.25, p<0.05), SANS-alogia (r=-0.29, p<0.01) and SANS-anhedonia (r=-0.16, p<0.05)	Average

Vargas et al. 2018<br><br>cross-sectional	UHR= 45 (SIPS);<br>mean age=18.9y;<br>Gender (n. males)= 24<br>Mean education= N/A	Neuroleptic-free= 41<br>SGA=4<br>CPZe=152.1	SIPS:<br>Social Anhedonia;<br>Avolition;<br>Expression of emotion;<br>Expressions of emotions and self;<br>Ideational richness;<br>Occupational functioning	Verbal memory=verb generation task	Selection costs of the verb generation task were positively associated with disorganized symptom score (r=0.409, p=0.01) and negative symptom score (r=0.38, p=0.01) and particularly with social anhedonia (r=0.29, p=0.03), avolition (r=0.5, p<0.001), ideational richness (r=0.3, p=0.025).	Poor

BLIPS= Brief Limited Intermittent Psychotic Symptoms; **BSIP**= Basel Screening Instrument for Psychosis; **BVMT**= Brief Visuospatial Memory Task; **CAARMS**= Comprehensive Assessment of At-Risk Mental States; **COPS** = Criteria of psychosis-risk syndrome**; CPT**=Continuous Performance Test; **CVLT**=California Verbal Learning Test; **DSB**=Digit Span Backword; **DSC**= Digital Symbol Coding subtest; **ER40**= Penn Emotion Recognition Task; **EDF40**= Penn Emotion Differentiation Task; **EXP**= Expressive Deficit domain of Negative Symptoms; **GF =** Global functioning scale; **HVLT**= Hopkins Verbal Learning Test; **HVLT-R**= Hopkins Verbal Learning Test – Revised Version; **IED=** Intra/Extra Dimensional Set Shift; **LNS**=Letter-Number Span; **LPS=** Leistungsprüfungssystem; **MAP**= Motivational Deficit Domain of Negative Symptoms; **MCCB** = Matrics Consensus Cognitive Battery; **MSCEIT**= Mayer-Salovey-Caruso Emotional Intelligence Test; **MWT-A=** Mehrfachwahl-Wortschatz-Test; **NAB**= Neuropsychological Assessment Battery; **NS**= Negative Symptoms; **PNS**= Primary Negative Symptoms; **PQ**= Prodomal Questionnaire; **RAD**= Relationship Across Domains; **RAVLT**=Rey Auditory Verbal Learning Test; **SANS**= Scale for the Assessment of Negative Symptoms; **SCWT**=Stroop Color and Word Test; **SCZ**=Patients with a diagnosis of schizophrenia; **SCZ-Aff=** Schizoaffective disorder; **SCZ-f**= schizophreniform disorder; **SFRT**= Situational Features Recognition Test**; SGA**=Second Generation Antipsychotic; **SIPS**= Structured Interview for Prodromal Syndromes; **SOFAS**= Social and Occupational Functioning Assessment Scale; **SOPS**= Scales Of Prodromal Symptoms; **TAP-WM**= Test of Attentional Performance, WM subtest; **TASIT**= The Awareness of Social Inference Test; **TMT**=Trail Making Test; **VPT**= Visual pattern test;

Table S3. Neurocognitive and Social Cognition assessment tools adopted in the included studies

**Neurocognitive composite score**	BACS	Bliksted et al. 2017<br>Glenthøj et al. 2017
	MCCB	Ventura et al. 2015
**Processing Speed**	Stroop test	Huang et al. 2016<br>Üçok et al. 2021<br>Buck et al. 2020
	TMT	Huang et al. 2016<br>Trampush et al. 2015<br>Stouten et al. 2017

		Shin et al. 2016<br>Rodríguez-Sánchez et al. 2008<br>Üçok et al. 2021<br>Lindgren et al. 2010b<br>Buck et al. 2020
	DSST	Meyer et al. 2014<br>Chang et al. 2020a<br>Wong et al. 2021<br>Lindgren et al. 2010b<br>Buck et al. 2020
	WAIS III Digit symbol subtest	Engen et al. 2019
	D-KEFS	Engen et al. 2019
	DSC	Glenthøj et al. 2017<br>Trampush et al. 2015
	MCCB	Ventura et al. 2015<br>Gerritsen et al. 2020
**Verbal fluency**	COWA	Meyer et al. 2014<br>Ayres et al. 2007
	letter and semantic fluency test	Shin et al. 2016
	Category verbal fluency	Chang et al. 2014<br>Chang et al. 2017<br>Stouten et al. 2017<br>Leanza et al. 2018b
	MCWST	Chang et al. 2020a<br>Wong et al. 2021
	D-KEFS	Engen et al. 2019
	Animal naming test	Hegde et al. 2013<br>Gerritsen et al. 2020
	FAS	Rodríguez-Sánchez et al. 2008
**Attention/Vigilance**	Choice reaction test	Saleem et al. 2013

	Digit Span and Letter Number sequencing test	Engen et al. 2019
	Digit span forward	Faerden et al. 2009
	TMT-A	Buck et al. 2020
	Brief test of attention	Rodríguez-Sánchez et al. 2008
	MCCB	Trampush et al. 2015<br>Ventura et al. 2015<br>Gerritsen et al. 2020
	Digit vigilance test	Hegde et al. 2013
	Continuous Performance test (sustained + selective)	Huang et al. 2016<br>Rodríguez-Sánchez et al. 2008<br>Stouten et al. 2017<br>Chan et al. 2006
	Color trails test	Hegde et al. 2013
	Stroop test	Üçok et al. 2021<br>Buck et al. 2020
	Go/No-Go Test (GNG)	Leanza et al. 2018b
	Triads test	Hegde et al. 2013
**Working Memory**	WAIS-R digit span subtest	Chang et al. 2016<br>Chang et al. 2017<br>Chang et al. 2020a<br>Wong et al. 2021<br>Shin et al. 2016
	WAIS-III	Üçok et al. 2021<br>Stouten et al. 2017<br>Shin et al. 2016
	MCCB	Ventura et al. 2015<br>Gerritsen et al. 2020<br>Trampush et al. 2015

	LNS	Faerden et al. 2009
	SOC	Saleem et al. 2013
	TAP	Leanza et al. 2018b
	Verbal N-back-task	Hegde et al. 2013
	MCCB	Gerritsen et al. 2020
	LNS subtest	Stouten et al. 2017
**Verbal Learning and Memory**	RAVLT	Hegde et al. 2013<br>Rodríguez-Sánchez et al. 2008<br>Stouten et al. 2017<br>Üçok et al. 2021
	WMS (logical memory)	Engen et al. 2019
	HVLT	Trampush et al. 2015<br>Ventura et al. 2015<br>Gerritsen et al. 2020
	BACS	Glenthøj et al. 2017
	Logical Memory Immediate and Delayed Recall	Buck et al. 2020
	California verbal learning test-II	Engen et al. 2019<br>Leanza et al. 2018b
**Visual Learning and Memory**	ROCF	Hegde et al. 2013
	Visual reproduction subtests	Buck et al. 2020
	BVMT	Trampush et al. 2015<br>Stouten et al. 2017<br>Ventura et al. 2015<br>Gerritsen et al. 2020

**Executive Functions**	WCST	Chang et al. 2016<br>Huang et al. 2016<br>Shin et al., 2016<br>Üçok et al. 2021<br>Chan et al. 2006
	D-KEFS	Engen et al., 2019<br>Faerden et al. 2009
	Stroop Test	Chang et al., 2016<br>Üçok et al. 2021<br>Chan et al. 2006
	TMT-B	Shin et al., 2016<br>Buck et al., 2020<br>Chan et al. 2006
	MCCB	Gerritsen et al. 2020<br>Ventura et al. 2015
	BACS	Glenthøj et al. 2017<br>Bliksted et al. 2017
	Penn CNB	Gur et al. 2015
	Intra/Extra Dimensional Set Shift	Saleem et al. 2013
	NAB Mazes	Trampush et al. 2015<br>Ventura et al. 2015
**Reasoning and problem solving**	NAB Mazes	Gerritsen et al. 2020<br>Trampush et al. 2015
	WCST	Meyer et al. 2014
	Tower of London	Stouten et al. 2017
	Tower of Hanoi	Chan et al. 2006
**Social Cognition**	MSCEIT	Trampush et al. 2015<br>Gerritsen et al. 2020
	TASIT	Bliksted et al. 2017

	SCRT	Piskulic and Addington 2011
	RAD	Barbato et al. 2015
	DACOBS	Stouten et al. 2017
	ANT	Stouten et al. 2017
	Hinting task	Stouten et al. 2017
	TASIT	Barbato et al. 2015
	ANT	Ditlevsen, Simonsen, and Bliksted 2020
	ToM advanced task	Mazza et al. 2012
	SAT	Ventura et al. 2015
	FEIT<br>FEDT	Piskulic and Addington 2011
	ER40, EDF40	Barbato et al. 2015
	WAIS-III-picture arrangement	Stouten et al. 2017
	i-GEOPTE-social cognition/basic cognitive functions subscores	Pelizza et al. 2021

ANT= Animated Triangles Task; **BVMT**= Brief Visuospatial Memory Task; **DACOBS**= Davos Assessment of Cognitive Biases Scale; **DSC**= Digital Symbol Coding subtest; **DSST**=Digit symbol substitution test **D-KEFS=** Delis–Kaplan Executive Functioning System; **ER40**= Penn Emotion Recognition Task; **EDF40**= Penn Emotion Differentiation Task; **EXP**= Expressive Deficit domain of

Negative Symptoms; **FAS**= Fluency Test; **FEDT**= Face Emotion Discrimination Task; **HEN**= High Royds Evaluation of Negativity Scale; **HVLT**= Hopkins Verbal Learning Test; **HVLT-R**= Hopkins Verbal Learning Test – Revised Version; **LNS**=Letter-Number Span; **MCCB** = Matrics Consensus Cognitive Battery; **MSCEIT**= Mayer-Salovey-Caruso Emotional Intelligence Test; **NAB**= Neuropsychological Assessment Battery; **RAD**= Relationship Across Domains; **ROCF**= Rey Osterrieth complex figure test; **SAT**= Social Animation Task; **SCRT**= Social Cue Recognition Test; **SOC**= Stocking of Cambridge; **TAP-WM**= Test of Attentional Performance, WM subtest; **TASIT**= The Awareness of Social Inference Test; **TAP**= Test of Attentional Performance; **TMT**=Trail Making Test; **WCST**= Wisconsin Card Sorting Test; **WMS-R**=Wechsler Memory Scale-Revised.

References

Ayres, A. M., G. F. Busatto, P. R. Menezes, M. S. Schaufelberger, L. Coutinho, R. M. Murray, P. K. McGuire, T. Rushe, and M. Scazufca. 2007. 'Cognitive deficits in first-episode psychosis: a population-based study in São Paulo, Brazil', *Schizophr Res*, 90: 338-43.

Barbato, Mariapaola, Lu Liu, Kristin S. Cadenhead, Tyrone D. Cannon, Barbara A. Cornblatt, Thomas H. McGlashan, Diana O. Perkins, Larry J. Seidman, Ming T. Tsuang, Elaine F. Walker, Scott W. Woods, Carrie E. Bearden, Daniel H. Mathalon, Robert Heinssen, and Jean Addington. 2015. 'Theory of mind, emotion recognition and social perception in individuals at clinical high risk for psychosis: Findings from the NAPLS-2 cohort', *Schizophrenia Research: Cognition*, 2: 133-39.

Bliksted, Vibeke, Poul Videbech, Birgitte Fagerlund, and Chris Frith. 2017. 'The effect of positive symptoms on social cognition in first-episode schizophrenia is modified by the presence of negative symptoms', *Neuropsychology*, 31: 209-19.

Buck, G., K. M. Lavigne, C. Makowski, R. Joober, A. Malla, and M. Lepage. 2020. 'Sex Differences in Verbal Memory Predict Functioning Through Negative Symptoms in Early Psychosis', *Schizophr Bull*, 46: 1587-95.

Chan, R. C., E. Y. Chen, and C. W. Law. 2006. 'Specific executive dysfunction in patients with first-episode medication-naïve schizophrenia', *Schizophr Res*, 82: 51-64.

Chang, W. C., C. L. Hui, S. K. Chan, E. H. Lee, G. H. Wong, and E. Y. Chen. 2014. 'Relationship between diminished expression and cognitive impairment in first-episode schizophrenia: a prospective three-year follow-up study', *Schizophr Res*, 152: 146-51.

Chang, W. C., V. W. Y. Kwong, C. L. M. Hui, S. K. W. Chan, E. H. M. Lee, and E. Y. H. Chen. 2017. 'Relationship of amotivation to neurocognition, self-efficacy and functioning in first-episode psychosis: a structural equation modeling approach', *Psychol Med*, 47: 755-65.

Chang, W. C., C. S. M. Wong, P. C. F. Or, A. O. K. Chu, C. L. M. Hui, S. K. W. Chan, E. M. H. Lee, Y. N. Suen, and E. Y. H. Chen. 2020a. 'Inter-relationships among psychopathology, premorbid adjustment, cognition and psychosocial functioning in first-episode psychosis: a network analysis approach', *Psychol Med*, 50: 2019-27.

———. 2020b. 'Inter-relationships among psychopathology, premorbid adjustment, cognition and psychosocial functioning in first-episode psychosis: a network analysis approach', *Psychol Med*, 50: 2019-27.

Chang, Wing Chung, Charles Fu Chun Lau, Sherina Suet In Chan, Christy Lai Ming Hui, Sherry Kit Wa Chan, Edwin Ho Ming Lee, Jingxia Lin, and Eric Yu Hai Chen. 2016. 'Premorbid, clinical and cognitive correlates of primary negative symptoms in first-episode psychosis', *Psychiatry Res*, 242: 144-49.

Ditlevsen, Jonas Vennike, Arndis Simonsen, and Vibeke Fuglsang Bliksted. 2020. 'Predicting mentalizing deficits in first-episode schizophrenia from different subdomains of negative symptoms', *Schizophr Res*, 215: 439-41.

Engen, Magnus Johan, Carmen Simonsen, Ingrid Melle, Ann Færden, Siv Hege Lyngstad, Beathe Haatveit, Anja Vaskinn, and Torill Ueland. 2019. 'Cognitive functioning in patients with first-episode psychosis stratified by level of negative symptoms: A 1-year follow-up study', *Psychiatry Res*, 281: 112554.

Faerden, A., A. Vaskinn, A. Finset, I. Agartz, E. Ann Barrett, S. Friis, C. Simonsen, O. A. Andreassen, and I. Melle. 2009. 'Apathy is associated with executive functioning in first episode psychosis', *BMC Psychiatry*, 9: 1.

Gerritsen, C., M. Maheandiran, J. Lepock, S. Ahmed, M. Kiang, R. M. Bagby, and R. Mizrahi. 2020. 'Negative symptoms in the clinical high-risk state for psychosis: Connection with cognition and primacy in impacting functioning', *Early Interv Psychiatry*, 14: 188-95.

Glenthøj, L. B., J. R. Jepsen, C. Hjorthøj, N. Bak, T. D. Kristensen, C. Wenneberg, K. Krakauer, M. Nordentoft, and B. Fagerlund. 2017. 'Negative symptoms mediate the relationship between neurocognition and function in individuals at ultrahigh risk for psychosis', *Acta Psychiatr Scand*, 135: 250-58.

Gur, R. E., M. March, M. E. Calkins, L. Weittenhiller, D. H. Wolf, B. I. Turetsky, and R. C. Gur. 2015. 'Negative symptoms in youths with psychosis spectrum features: complementary scales in relation to neurocognitive performance and function', *Schizophr Res*, 166: 322-7.

Hegde, S., J. Thirthalli, S. L. Rao, A. Raguram, M. Philip, and B. N. Gangadhar. 2013. 'Cognitive deficits and its relation with psychopathology and global functioning in first episode schizophrenia', *Asian J Psychiatr*, 6: 537-43.

Huang, M., Y. Huang, L. Yu, J. Hu, J. Chen, P. Jin, W. Xu, N. Wei, S. Hu, H. Qi, and Y. Xu. 2016. 'Relationship between negative symptoms and neurocognitive functions in adolescent and adult patients with first-episode schizophrenia', *BMC Psychiatry*, 16: 344.

Leanza, L., L. Egloff, E. Studerus, C. Andreou, U. Heitz, S. Ittig, K. Beck, M. Uttinger, and A. Riecher-Rössler. 2018a. 'The relationship between negative symptoms and cognitive functioning in patients at clinical high risk for psychosis', *Psychiatry Res*, 268: 21-27.

Leanza, Letizia, Laura Egloff, Erich Studerus, Christina Andreou, Ulrike Heitz, Sarah Ittig, Katharina Beck, Martina Uttinger, and Anita Riecher-Rössler. 2018b. 'The relationship between negative symptoms and cognitive functioning in patients at clinical high risk for psychosis', *Psychiatry Res*, 268: 21-27.

Lee, E. H. M., C. L. M. Hui, K. P. K. Chan, P. Y. Chan, E. Y. L. Law, C. S. Y. Chong, W. C. Chang, S. K. W. Chan, W. K. Lee, A. W. F. Lo, E. P. F. Pang, E. F. C. Cheung, M. G. C. Yiu, D. W. S. Chung, R. M. K. Ng, W. S. Yeung, W. T. L. Lo, and E. Y. H. Chen. 2019. 'The role of symptoms and insight in mediating cognition and functioning in first episode psychosis', *Schizophr Res*, 206: 251-56.

Lindgren, M., M. Manninen, T. Laajasalo, U. Mustonen, H. Kalska, J. Suvisaari, K. Moilanen, T. D. Cannon, M. Huttunen, and S. Therman. 2010a. 'The relationship between psychotic-like symptoms and neurocognitive performance in a general adolescent psychiatric sample', *Schizophr Res*, 123: 77-85.

Lindgren, Maija, Marko Manninen, Taina Laajasalo, Ulla Mustonen, Hely Kalska, Jaana Suvisaari, Kari Moilanen, Tyrone D. Cannon, Matti Huttunen, and Sebastian Therman. 2010b. 'The relationship between psychotic-like symptoms and neurocognitive performance in a general adolescent psychiatric sample', *Schizophr Res*, 123: 77-85.

Mazza, M., R. Pollice, F. Pacitti, M. C. Pino, M. Mariano, S. Tripaldi, M. Casacchia, and R. Roncone. 2012. 'New evidence in theory of mind deficits in subjects with chronic schizophrenia and first episode: correlation with symptoms, neurocognition and social function', *Riv Psichiatr*, 47: 327-36.

Meyer, E. C., R. E. Carrión, B. A. Cornblatt, J. Addington, K. S. Cadenhead, T. D. Cannon, T. H. McGlashan, D. O. Perkins, M. T. Tsuang, E. F. Walker, S. W. Woods, R. Heinssen, and L. J. Seidman. 2014. 'The relationship of neurocognition and negative symptoms to social and role functioning over time in individuals at clinical high risk in the first phase of the North American Prodrome Longitudinal Study', *Schizophr Bull*, 40: 1452-61.

Niendam, Tara A., Carrie E. Bearden, Jennifer K. Johnson, Malin McKinley, Rachel Loewy, Mary O'Brien, Keith H. Nuechterlein, Michael F. Green, and Tyrone D. Cannon. 2006. 'Neurocognitive performance and functional disability in the psychosis prodrome', *Schizophrenia Research*, 84: 100-11.

Pelizza, L., D. Maestri, E. Leuci, E. Quattrone, S. Azzali, G. Paulillo, and P. Pellegrini. 2021. 'Negative symptom configuration in patients with first episode affective psychosis: findings from the 1-year follow-up of the "Parma Early Psychosis" program', *Acta Biomed*, 92: e2021224.

Piskulic, Danijela, and Jean Addington. 2011. 'Social cognition and negative symptoms in psychosis', *Psychiatry Res*, 188: 283-85.

Rodríguez-Sánchez, J. M., B. Crespo-Facorro, C. González-Blanch, R. Pérez-Iglesias, M. Alvarez-Jiménez, O. Martínez, and J. L. Vázquez-Barquero. 2008. 'Cognitive functioning and negative symptoms in first episode schizophrenia: different patterns of correlates', *Neurotox Res*, 14: 227-35.

Saleem, Majid M, Michael K Harte, Kay M Marshall, Andy Scally, Anita Brewin, and Jo C Neill. 2013. 'First episode psychosis patients show impaired cognitive function – a study of a South Asian population in the UK', 27: 366-73.

Shin, Y. S., S. Y. Kim, T. Y. Lee, J. W. Hur, N. Y. Shin, S. N. Kim, M. S. Shin, and J. S. Kwon. 2016. 'Longitudinal change in neurocognition and its relation to symptomatic and functional changes over 2years in individuals at clinical high-risk for psychosis', *Schizophr Res*, 174: 50-57.

Stouten, L. H., W. Veling, W. Laan, M. van der Helm, and M. van der Gaag. 2017. 'Psychosocial functioning in first-episode psychosis and associations with neurocognition, social cognition, psychotic and affective symptoms', *Early Interv Psychiatry*, 11: 23-36.

Trampush, J. W., T. Lencz, P. DeRosse, M. John, J. A. Gallego, G. Petrides, Y. Hassoun, J. P. Zhang, J. Addington, C. H. Kellner, M. Tohen, K. E. Burdick, T. E. Goldberg, J. M. Kane, D. G. Robinson, and A. K. Malhotra. 2015. 'Relationship of Cognition to Clinical Response in First-Episode Schizophrenia Spectrum Disorders', *Schizophr Bull*, 41: 1237-47.

Üçok, A., N. Direk, H. Kaya, N. Çağlar, U. Çıkrıkçılı, H. Noyan, Ç Yokuşoğlu, and M. Devrim-Üçok. 2021. 'Relationship of negative symptom severity with cognitive symptoms and functioning in subjects at ultra-high risk for psychosis', *Early Interv Psychiatry*, 15: 966-74.

Vargas, T., H. Snyder, M. Banich, R. Newberry, S. A. Shankman, G. P. Strauss, and V. A. Mittal. 2018. 'Altered selection during language processing in individuals at high risk for psychosis', *Schizophr Res*, 202: 303-09.

Ventura, J., A. Ered, D. Gretchen-Doorly, K. L. Subotnik, W. P. Horan, G. S. Hellemann, and K. H. Nuechterlein. 2015. 'Theory of mind in the early course of schizophrenia: stability, symptom and neurocognitive correlates, and relationship with functioning', *Psychol Med*, 45: 2031-43.

Wong, S. C. Y., W. C. Chang, C. L. M. Hui, S. K. W. Chan, E. H. M. Lee, Y. N. Suen, and E. Y. H. Chen. 2021. 'Relationship of subjective quality of life with symptomatology, neurocognition and psychosocial functioning in first-episode psychosis: a structural equation modelling approach', *Eur Arch Psychiatry Clin Neurosci*, 271: 1561-69.
